# Global adoption of personal and social mitigation behaviors during COVID-19: The role of trust & confidence

**DOI:** 10.1371/journal.pone.0256159

**Published:** 2021-09-08

**Authors:** Pauline Jones, Anil Menon, Allen Hicken, Laura S. Rozek

**Affiliations:** 1 Department of Political Science, College of Literature, Science, and Arts, University of Michigan -Ann Arbor, Ann Arbor, Michigan, United States of America; 2 Department of Environmental Health Sciences, Nutrition, and Global Public Health, School of Public Health, University of Michigan—Ann Arbor, Ann Arbor, Michigan, United States of America; VIT University, INDIA

## Abstract

What influences the adoption of SARS-CoV-2 mitigation behaviors–both personal, such as mask wearing and frequent handwashing, and social, such as avoiding large gatherings and physical contact–across countries? Understanding why some individuals are more willing to change their behavior to mitigate the spread of a pandemic will not only help us to address the current SARS-CoV-2 pandemic but also to respond to future ones. Researchers have pointed to a variety of factors that may influence individual adoption of personal and social mitigation behaviors, including social inequality, risk perception, personality traits, and government policies. While not denying the importance of these factors, we argue that the role of trust and confidence has received insufficient attention to date. Our study explores whether there is a difference in the way trust and confidence in particular leaders and organizations affect individual compliance and whether this effect is consistent across different types of mitigation behaviors. Specifically, we utilize an original cross-national survey conducted during the first wave of the SARS-CoV-2 pandemic (May-June 2020) to investigate how trust in scientists, medical professionals, politicians, and religious leaders and confidence in global, national, and local health organizations affects individual compliance in 16 countries/territories across five world regions. Our analyses, which control for the aforementioned factors as well as several others, suggest that trust in politicians and confidence in national health ministries have the most consistent influence on whether individuals adopt both personal and social mitigation behaviors. Across our sample, we find that greater trust in politicians is associated with lower levels of individual compliance with public health directives, whereas greater confidence in the national health ministry is associated with higher levels of individual compliance. Our findings suggest the need to understand trust and confidence as among the most important individual level characteristics driving compliance when developing and delivering messaging about the adoption of mitigation behaviors. The content of the message, it seems, will be most effective when citizens across countries trust its source. Trusted sources, such as politicians and the national health ministry, should thus consider working closely together when determining and communicating recommended health behaviors to avoid contradicting one another.

## Introduction

The gradual rollout of vaccines targeting the SARS-CoV-2 virus promises a slow but sure return to normalcy. In the meantime, continued conformance with certain changes in our health behavior will be key to mitigating the spread of the virus. These changes include both personal behaviors, such as mask wearing and frequent handwashing, and social distancing behaviors, such as avoiding large gatherings and physical contact. It is also increasingly clear that the pandemic cannot be contained if these behaviors are not widely adopted–that is, the adoption of mitigation behaviors is a shared responsibility both within and across countries [1]. In addition, the willingness of individuals across countries to quickly adopt mitigation behaviors will be key to minimizing the fallout from future pandemics. Several studies have analyzed the extent to which people adopt recommended and required behaviors in response to previous coronavirus and influenza pandemics, such as H1N1 and SARS-CoV, in individual countries [2–4]. However, there has been little effort to identify common factors across countries that might influence the extent to which people in different countries comply with government policies and recommendations regarding mitigation behaviors.

Social and behavioral scientists have pointed to a myriad of factors that may affect the extent to which individuals are willing and able to adopt personal and social distancing mitigation behaviors [5]. Among the most prominent are social inequality, risk perception, personality traits, and government policies. Social inequalities due to differences in socio-economic status (SES) are expected not only to increase the likelihood of exposure to the virus but also to decrease compliance with recommended behaviors that would reduce exposure because many individuals have insufficient resources to do so [6, 7]. Conversely, greater risk perception–which can be related to the fear of being exposed personally to the virus or of one’s family contracting the virus [8, 9]–is expected to increase compliance with government policies and recommendations [10]. Individuals with certain personality traits (i.e., the “Big Five”–extroversion, agreeableness, conscientiousness, neuroticism and openness) are also expected to be more compliant, and cross-national research on COVID-19 has shown that all except extroversion are associated with adopting recommended mitigation behaviors [11, 12]. Finally, regarding the effectiveness of stricter government social distancing measures, research to date on compliance with SARS-CoV-2 related policies has produced mixed results [13].

While not denying the importance of these factors, we argue that the role that trust and confidence play in shaping individual adoption of mitigation behaviors warrants greater attention. Previous studies have found both that trust in political leaders and confidence in healthcare providers are in decline and that they are crucial to individual compliance with health directives [14, 15]. Trust and confidence are deemed to be particularly important for compliance, moreover, when people are being asked to make personal sacrifices for the greater good because they need to believe that the social benefits are worth the individual cost [15, 16]. Trust and confidence should also be crucial in the context of a crisis, such as the SARS-CoV-2 pandemic, because most people lack the knowledge and expertise to make decisions about either the degree of risk or the best course of action to mitigate that risk [17, 18]. Yet, our knowledge remains limited because most studies to date rely on single country data or focus exclusively on developed countries [19] and do not explore how trust and confidence in different types of leaders and organizations might affect compliance with different types of mitigation behaviors. Existing studies also tend to assume that the relationship between trust and individual compliance is positive, without differentiating among objects of that trust [20].

We investigate how trust in several key domestic actors, including scientists, medical professionals, politicians, and religious leaders, and confidence in global, national, and local health organizations affects individual compliance across 16 countries/territories in five world regions. Our analyses, which control for social inequality, risk perception, the Big Five personality traits, and government policies, as well as other important factors, suggest that trust in politicians and confidence in national health ministries have the most consistent influence on the adoption of both personal and social distancing mitigation behaviors. However, trust is not uniformly associated with better compliance. We find that greater trust in politicians is associated with lower levels of individual compliance with public health directives, whereas greater confidence in the national health ministry is associated with higher levels of individuals compliance.

## Methods

### Hypotheses

We use data from an original cross-national survey (described below) to test four hypotheses (H1-H4) concerning the relationship between confidence and trust and both personal and social mitigation behaviors during the SARS-CoV-2 pandemic. First, consistent with previous studies [21], we expect both higher levels of trust in scientists and medical practitioners and confidence in healthcare organizations at all levels to be positively associated with voluntary compliance with public health directives (H1 and H2). Trust in healthcare professionals and institutions is widely known to have a positive effect on individuals willingness to follow health protocols under normal circumstances [22, 23]. This relationship is likely to be intensified during a health crisis. In the context of the Ebola epidemic, for example, efforts to build trust in local health officials were successful in promoting the adoption of recommended health behaviors [24]. Likewise, research on the adoption of recommended health behaviors during H1N1 influenza pandemic suggests that trust in the national health ministry increases the likelihood of individual compliance [25]. More recent research has found that trust in science and confidence in the domestic healthcare system are highly correlated with the adoption of SARS-CoV-2 prevention behaviors in European countries [26]. Related research, moreover, suggests that confidence in both domestic and international health organizations is strongly associated with vaccine acceptance during the SARS-CoV-2 pandemic among adults across multiple countries [27].

Second, contrary to much of the existing literature, we expect greater trust in politicians to be negatively associated with voluntary compliance with public health directives. Although the general consensus is that higher levels of political trust are positively associated with individual compliance [20], findings from previous studies are mixed. Some single-country studies have found that high levels of political trust were associated with compliance concerning both health directives in general and social distancing policies in particular during Ebola [28]. Meanwhile, other cross-national studies have found that trust in government is not positively associated with individual adoption of recommended health behaviors during SARS-CoV-2 [29]. This scenario is easiest to imagine where anti-science political leaders are in direct conflict with health officials (most notably in the U.S., Brazil, the Philippines, and India) [30]. However, we argue that even where politicians are not overtly challenging the advice of health professionals, trust in politicians is likely to have a negative effect on individual compliance because incumbents have incentives to underplay the severity of a pandemic, and thus, the need for adopting mitigation behaviors (H3). In the context of SARS-CoV-2, several factors directly contributed to these incentives, including the political costs attached with the managing of a public health crisis and the potentially severe economic consequences of pandemic mitigation [31]. Of course, it is possible that there is no relationship between trust in politicians and mitigation behaviors, either because politicians are not salient actors for individual-level mitigation decision making, or because politicians within a country take different positions and thus to do not send a clear signal to citizens. To the extent that either of these situations applies, this should bias our results towards the null hypothesis of no association between trust and mitigation behavior.

Finally, we expect to find a negative association between greater trust in religious leaders and the adoption of social distancing guidelines but no association in either direction with the adoption of personal mitigation behaviors (H4). Existing research on religion and compliance has focused mostly on religiosity, finding a negative relationship with the willingness to adopt mitigation behaviors, but it does not provide much insight into the role of trust in religious leaders [32]. Yet, the literature on religious leaders suggests that they influence individual attitudes about salient social and political issues [33] as well as a range of important social and political behaviors [34]. This literature also suggests that their influence may be magnified in times of crisis, such as a pandemic, when people are more likely to turn to religion because they face elevated levels of threat and uncertainty [35]. We argue that both for this reason and due to the communal nature of religious services and holiday celebrations, religious leaders are often motivated to advise their congregations not to comply with social distancing guidelines. In other words, the SARS-CoV-2 pandemic provided religious leaders with an opportunity both to reassure their existing congregants and to expand their congregation to include those newly seeking comfort in religion. Advising congregants not to attend services or celebrations would run counter to these incentives.

H1: Greater confidence in global, national, and local health organizations is positively associated with the adoption of personal and social distancing mitigation behaviors.
H2: Greater trust in medical practitioners and scientists is positively associated with the adoption of personal and social distancing mitigation behaviors.
H3: Greater trust in politicians is negatively associated with the adoption of personal and social distancing mitigation behaviors.
H4: Greater trust in religious leaders is negatively associated with the adoption of social distancing mitigation behaviors.

### Study design/data collection

We conducted an online cross-sectional survey in sixteen countries/territories across five world regions: North America (Canada, United States); Europe/Eurasia (Germany, Poland, Sweden, Ukraine); East Asia (China, Hong Kong, Taiwan); Southeast Asia (Indonesia, Malaysia, Philippines, Singapore, Thailand, Vietnam); and the Middle East (Turkey). In order to maximize the diversity of our sample, we included countries and territories in our survey that varied across several important factors–their level of economic development, dominant culture, regime type, and government response to the pandemic–while also taking into consideration the area expertise of our research team. The survey was translated into the respective local languages for each country/territory. For all sixteen countries/territories included in the analysis, we used the Qualtrics online survey platform due to the constraints against conducting in-person surveys in the context of a pandemic. Qualtrics maintains a country-level database of residents who have volunteered to participate in survey-based research from which Qualtrics recruits survey respondents via Qualtrics panels, enabling us to achieve high response rates (www.qualtrics.com). Panel research is a rapid method for collecting data repeatedly, drawing a sample from a pre-recruited set of respondents. We used quota sampling methods to target a Qualtrics panel sample that was representative of the country’s/territory’s demographics with respect to age and gender. This approach is in accordance with the policies of the Institutional Review Board at the University of Michigan. Of the 35,960 people who accessed the Qualtrics landing page and reviewed the consent form, 16,708 (46.5%) completed the survey. Data were collected between May 21 and June 24, 2020.

This study was reviewed by the Health Sciences and Behavioral Sciences Institutional Review Board (IRB-HBHS) at the University of Michigan and categorized as exempt (HUM00168677). All participants in the study were required to give informed consent, in accordance with the policies of the IRB-HBHS, before they could access the survey via Qualtrics. All participants were compensated via Qualtrics according to their guidelines. The rate for a 25-minute survey usually ranges from $3–4 US dollars.

### Mitigation behaviors

To assess compliance at the individual level, respondents were asked if they engaged in a range of personal and social mitigation behaviors. This article focuses on three measures of these self-reported mitigation behaviors. The first two dependent variables map on to individual best practices for reducing the spread of the virus that were most widely recommended by health experts and national governments: wearing masks and washing hands frequently. Individuals who reported adopting these behaviors were given a value of 1, 0 otherwise. The third dependent variable is a measure of social distancing that we constructed by combining information on respondent adoption (or lack thereof) of 3 behaviors that could impose a social cost on respondents–avoiding social events, avoiding enclosed spaces outside one’s home, and avoiding physical contact when greeting friends and family members. Individuals who reported adopting all 3 behaviors received a score of 3, while those who acknowledged adopting either 2 or one of these mitigation practices received a score of 2 and 1, respectively. Individuals who reported that they did not follow any of these social distancing practices were given a score of 0.

### Trust and confidence

While there are subtle conceptual distinctions between confidence and trust [36], international surveys generally use the two terms interchangeably, and we adopt a similar approach here. Specifically, we follow the model set by the internationally renowned World Values Survey in using “confidence” when referring to organization and "trust" when referring to individual actors. Respondents were asked about confidence in global, national, and local health organizations and trust in several key domestic actors. To assess confidence, respondents were asked how much confidence they had in the ability of the WHO, their national health organizations, and local health departments to handle the SARS-CoV-2 pandemic. They could choose among the following 4 options: no confidence at all, not very much confidence, some confidence, and a lot of confidence. Regarding scientists, medical practitioners, religious leaders, and politicians, respondents were asked to report their levels of general trust in each of these groups. Unlike health organizations, we did not specify at which level (i.e., local, national, etc.) these various leaders operate so as not to limit the scope of respondents’ assessment to a particular set of individuals. Concerning politicians, for example, this is important because it allows us to avoid conflating political trust with trust in a specific political figure [15]. Respondents could choose one of the following 4 options: do not trust at all, do not trust very much, trust somewhat, and trust completely. (Please refer to S1 and S2 Figs for the distribution of these variables by country/territory.)

### Risk perception

In order to measure risk perception, we asked respondents to report two types of perceived risk: first, the extent to which they were concerned that they themselves would be infected with SARS-CoV-2; and second, the extent to which they were concerned that their loved ones would be infected with SARS-CoV-2. Respondents were given the following response options: not at all, somewhat, very much, and extremely.

### Personality traits

We also include a measure for each of the Big Five personality traits. Specifically, we asked respondents the extent to which they see themselves in the following ways: 1) Extroverted and/or enthusiastic (extroversion); 2) Critical and/or quarrelsome (agreeableness) 3) Dependable and/or self-disciplined (conscientiousness); 4) Anxious and/or easily upset (neuroticism); and 5) Open to new experiences and/or creative (openness). Respondents could choose one of the following options: disagree strongly, disagree a little, agree a little, or agree strongly.

### Government policies

We account for differences in government policies across countries in two ways. First, we control for objective differences by including country fixed effects, which is a commonly used statistical tool to adjust for differences across respondents that might result from their belonging to different countries (for example, the effect of being in Sweden, which did not require mask wearing, versus Germany, which did). Second, we control for subjective differences by asking citizens directly which government policies have been enacted to reduce the spread of SARS-CoV-2. This approach has the additional benefit of avoiding the tendency to infer compliance from outcomes–that is, when policies are effective in reducing the spread of the virus there is a presumption that this is because individuals knowingly complied with these policies [13, 37]. Specifically, to examine whether the adoption of mitigation behaviors was driven by awareness of existing policies, we asked respondents whether their governments had enacted the following mandates at the time of the survey: restricting gatherings to a small number of people, restricting gatherings to people in one’s immediate household, and requiring face masks in all public places. They were provided the following response options: never in force, previously in force but not now, currently in force, and not sure. Individuals who responded that a guideline was currently in force were coded as 1, and 0 otherwise.

### Other controls

Respondents also provided information on their age (18–29, 30–49, 50–69, 70+), sex (male, female), education (less than high school, high school, some college or post-secondary education, 4-year college graduate, graduate or professional training beyond college, doctoral degree), country/territory of residence (Canada, China, Germany, Hong Kong, Indonesia, Malaysia, Philippines, Poland, Singapore, Sweden, Taiwan, Thailand, Turkey, Ukraine, United States, Vietnam), and socioeconomic situation prior to the implementation of any pandemic-relief policies (we do not have enough money for food; we have enough money for food, but not enough money for clothes; we have enough money to buy food and clothes, but not enough to buy expensive items, such as a TV or refrigerator; we have expensive items, such as a new TV or refrigerator, but no car; we can buy almost anything we want). Importantly, the latter variable was included because those who were more vulnerable economically might have been less able to follow mitigation guidelines.

### Analysis

We estimated the relationship between the confidence and trust measures and individual adoption of SARS-CoV-2 mitigation behaviors using logistic regressions. These multivariable models included age, education, socioeconomic status, sex, concern about self and loved ones contracting the virus, and perceptions regarding government guidelines in effect at the time of survey. Importantly, they also included country-fixed effects, which is a commonly used statistical tool to adjust for differences across respondents that might result from their belonging to different countries/territories. This enables us to estimate the relationship between trust and confidence and compliance with mitigation behaviors at the individual level without the interference of country-level characteristics. We conducted all our analyses using R statistical software (The R Foundation, 3.6.3). The total number of observations utilized in the regressions (14,184) is slightly lower than the number of respondents for each question because any respondent for whom a value is missing across any of the variables included in the analysis is dropped when estimating marginal effects.

## Results

**Fig 1:**
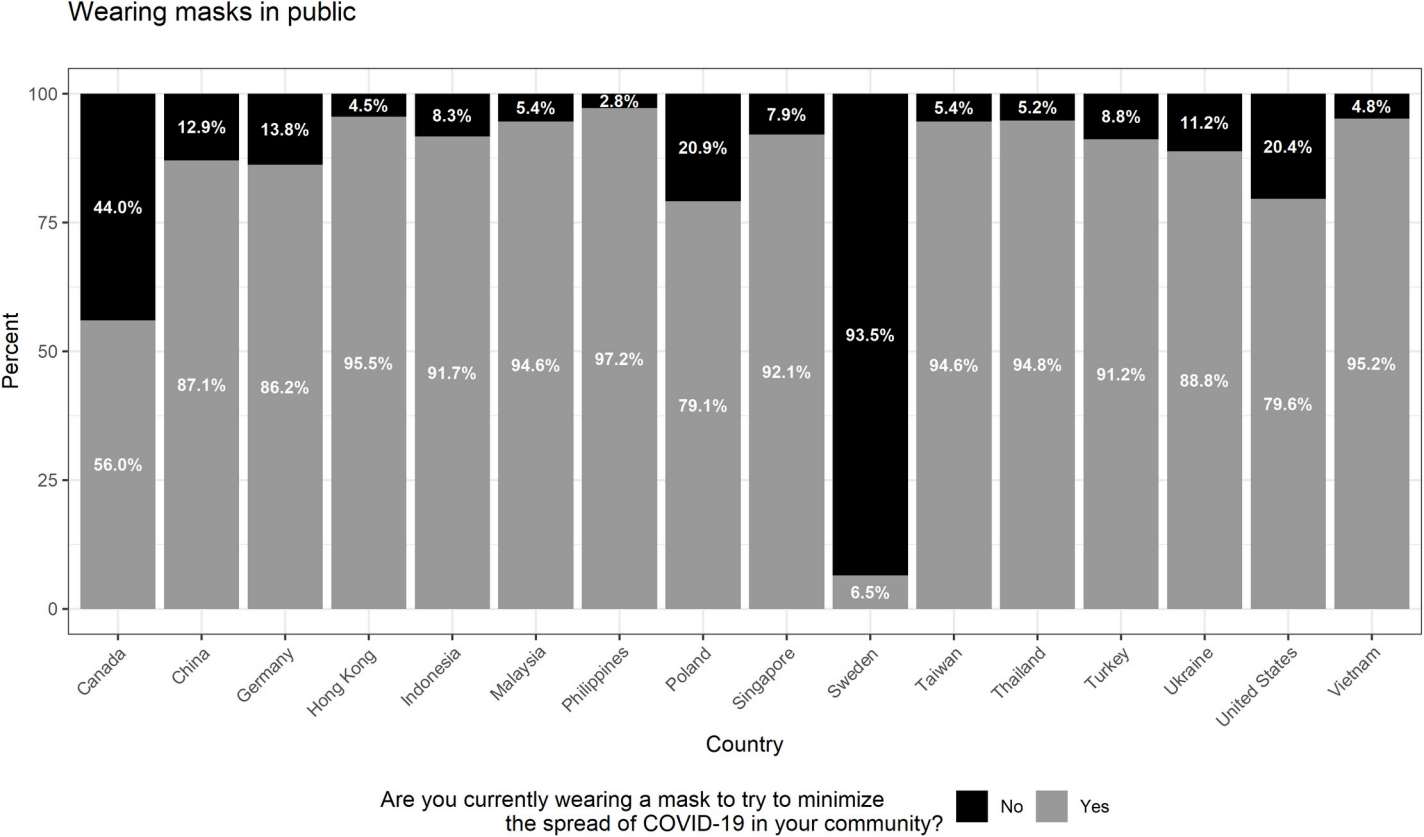
Self-reported mask wearing by country/territory. This figure summarizes individual responses (yes, no), by country/territory, regarding whether they have been wearing a face mask to try to minimize the spread of the COVID-19 in their community. Sample size by country/territory: Canada = 1077; China = 1177; Germany = 1056; Hong Kong = 582; Indonesia = 1097; Malaysia = 959; Philippines = 1160; Poland = 1107; Singapore = 541; Sweden = 1058; Taiwan = 759; Thailand = 1105; Turkey = 1108; Ukraine = 1101; United States = 1228; Vietnam = 1048.

**Fig 2:**
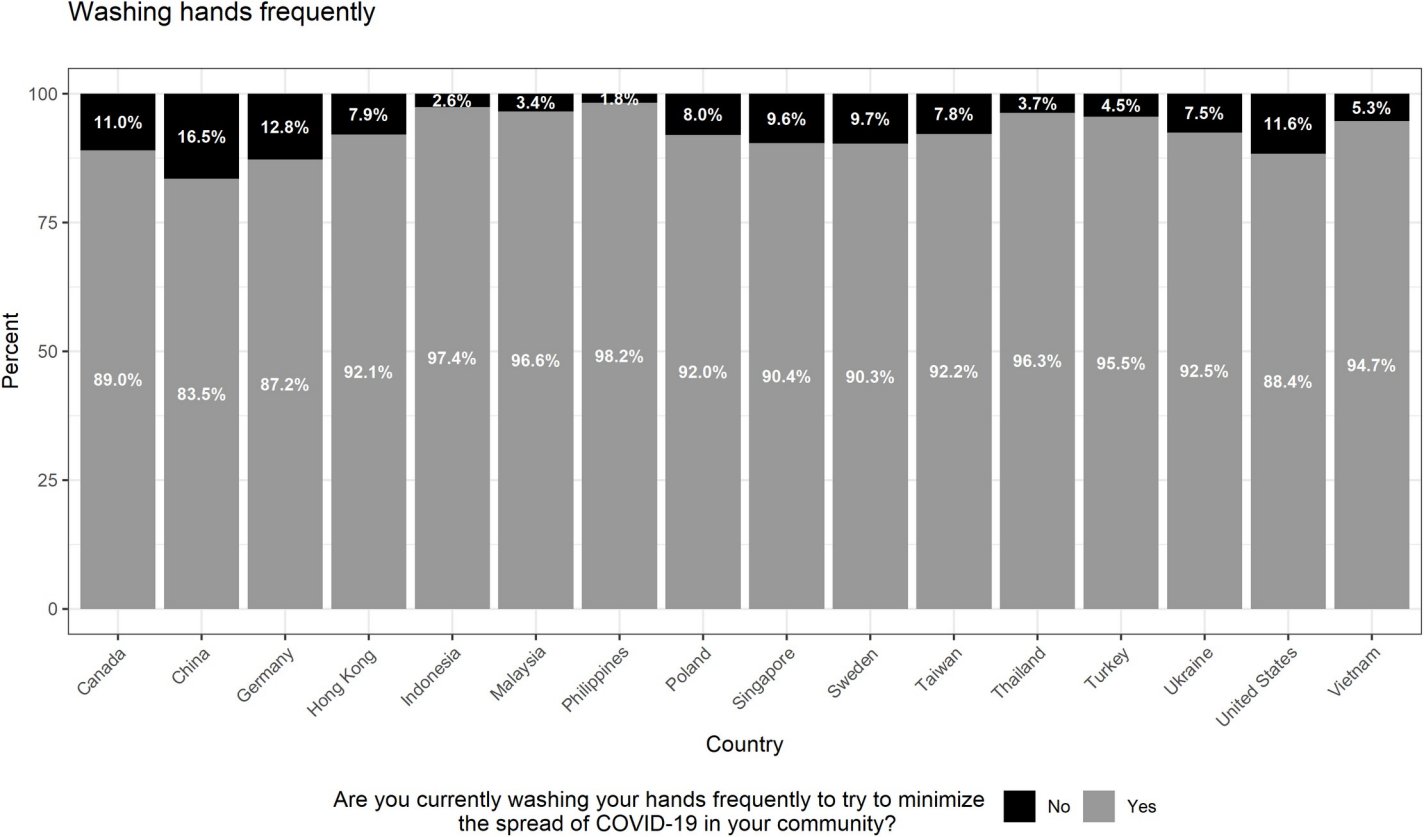
Self-reported hand washing by country/territory. This figure summarizes individual responses (yes, no), by country/territory, regarding whether they have been washing their hands more often to try to minimize the spread of the COVID-19 in their community. Sample size by country/territory: Canada = 1077; China = 1177; Germany = 1056; Hong Kong = 582; Indonesia = 1097; Malaysia = 959; Philippines = 1160; Poland = 1107; Singapore = 541; Sweden = 1058; Taiwan = 759; Thailand = 1105; Turkey = 1108; Ukraine = 1101; United States = 1228; Vietnam = 1048.

Our sample consists of 16,708 respondents across 16 countries/territories. (Summary statistics are presented in S1 Table.) A majority of respondents in our sample reported that they had adopted personal mitigation behaviors; specifically, 82% and 92% of respondents said they were wearing masks and washing their hands frequently, respectively, to mitigate the spread of the virus. Figs 1 and 2 show the distribution by country/territory. Notably, there was some variation by country/territory, though much more so for mask wearing than for handwashing. The proportion of respondents answering “yes” to whether they were wearing masks ranged from 6.5% (Sweden) to 95.2% (Vietnam) whereas the proportion of respondents answering “yes” to whether they were washing their hands ranged from 83.5% (China) to 98.2% (Philippines).

**Fig 3:**
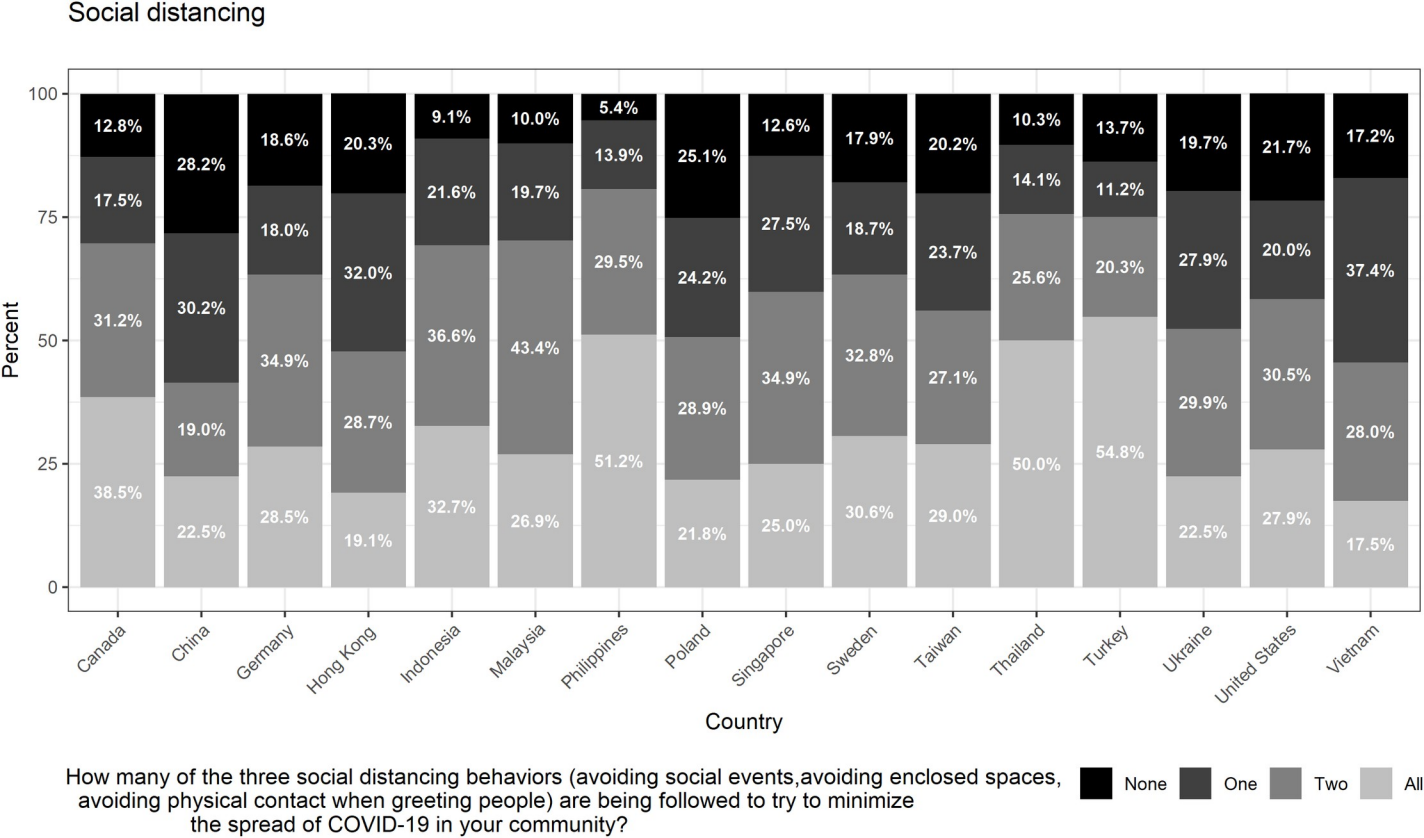
Self-reported social distancing by country/territory. This figure summarizes individual responses (no behaviors adopted, one behavior, two behaviors, all three behaviors), by country/territory, regarding how many of the three social distancing behaviors (avoiding social events, avoiding enclosed spaces, avoiding physical contact when greeting people) they are following to try to minimize the spread of COVID-19 in their community. Sample size by country/territory: Canada = 1077; China = 1177; Germany = 1056; Hong Kong = 582; Indonesia = 1097; Malaysia = 959; Philippines = 1160; Poland = 1107; Singapore = 541; Sweden = 1058; Taiwan = 759; Thailand = 1105; Turkey = 1108; Ukraine = 1101; United States = 1228; Vietnam = 1048.

Similarly, the vast majority of our respondents in our sample reported that they had adopted at least one of the three types of social distancing behaviors we include in our measure. Only 16% of respondents said that they had adopted none of the 3 types of behaviors, whereas 22%, 30%, and 32% of respondents stated that they had adopted one, 2, or all 3 of the social distancing behaviors. As depicted in Fig 3, the degree to which individuals are adopting social distancing behaviors varies significantly by country/territory. The proportion of respondents answering that they had adopted all three types of behaviors, for example, ranges from 54.8% (Turkey) to 17.5% (Vietnam).

**Fig 4:**
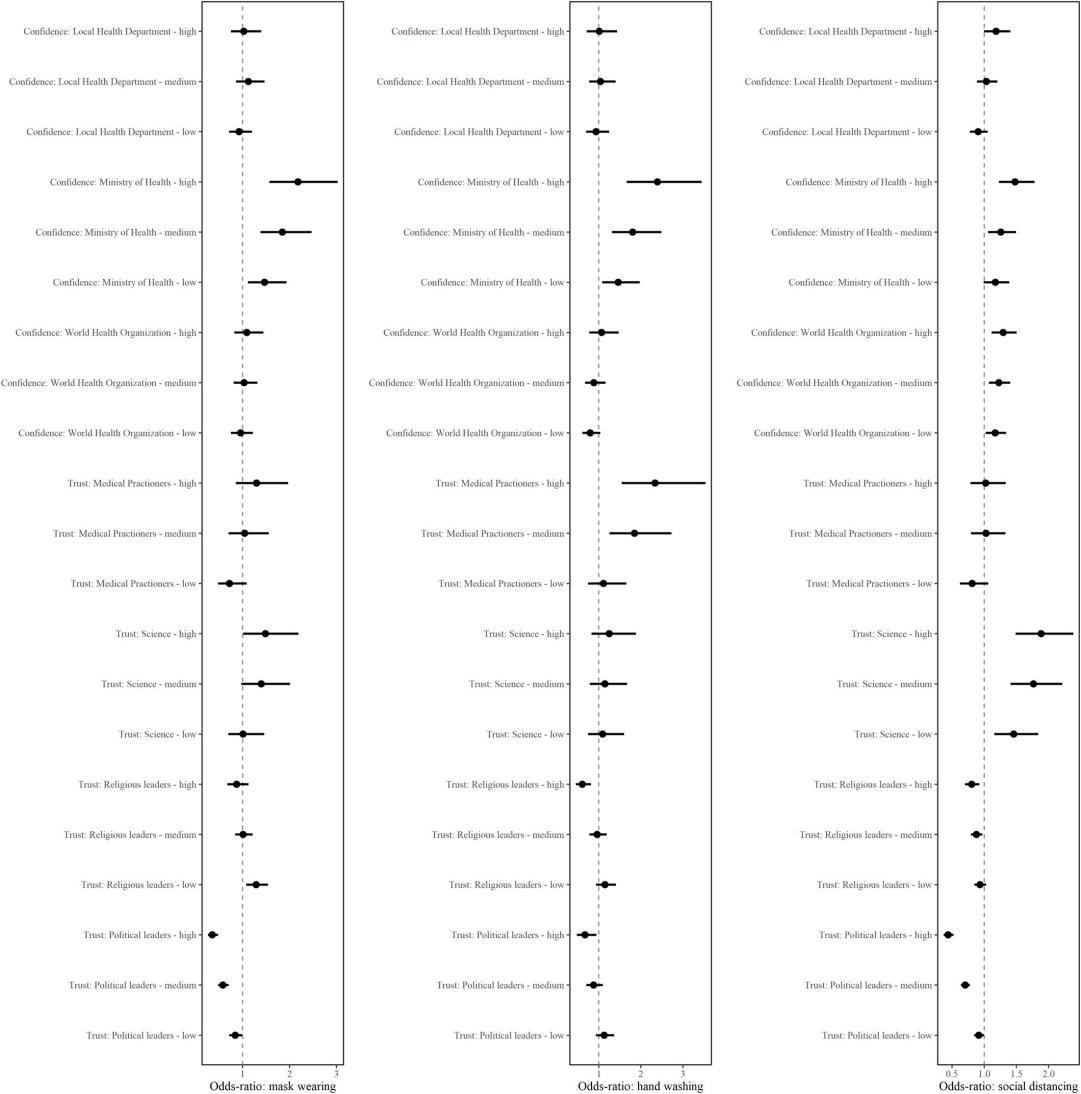
Multivariable modeling of confidence, trust, and adoption of mitigation behaviors. Results from estimating the relationship between confidence and trust measures and vaccine hesitancy using logistic regression. These multivariable models also include age, education, socioeconomic status, gender, risk perception, perception of government policies, personality traits, and country-fixed effects. Please refer to the Methods section of the paper for details on individual variables and to S2 Table for the full results from these specifications.

We now turn to the results from our multivariate analysis. Odds ratios (OR) and 95% confidence intervals are summarized in Fig 4. (Full results are presented in S2 Table.)

### Personal mitigation behaviors

For both types of personal mitigation behaviors–mask wearing and hand washing–we found a positive association with greater confidence in national health care organizations, a negative association with greater trust in politicians, and no statistically significant association with greater confidence in the WHO. There were also some important differences: mask wearing was positively associated with greater trust in scientists while hand washing was also positively associated with greater trust in medical practitioners.

Individuals who reported greater confidence in national health organizations to handle the pandemic and greater trust in scientists also reported increased mask wearing (Fig 4, Column 1). The odds that individuals who report medium and high levels of confidence in national health organizations report wearing masks to mitigate the spread of the virus are 85% (OR = 1.85, p < .001, 95% CI: 1.38, 2.47) and 118% (OR = 2.18, p < .001, 95% CI: 1.57, 3.03) higher compared to individuals reporting no trust in national health organizations. The odds that individuals who report high levels of trust in scientists have been wearing masks to mitigate the spread of the virus are higher by 49% (OR = 1.49, p < .05, 95% CI: 1.006, 2.18) compared to individuals reporting no trust in scientists. Meanwhile, individuals who reported more trust in politicians also reported decreased mask wearing (Fig 4, Column 1). The odds that individuals with no trust in politicians have been wearing masks to mitigate the spread of the virus are higher by 184% (OR = 2.84, p < .001, 95% CI: 2.10, 3.83) compared to individuals who report high levels of trust in politicians.

Both greater confidence in national health organizations and more trust in medical practitioners were positively associated hand washing among our respondents, but neither confidence in the WHO nor trust in scientists were associated with this behavior (Fig 4, Column 2). Compared to individuals who report no confidence in national health organizations, the odds that individuals who report medium and high levels of confidence in national health organizations also report hand washing to mitigate the spread of the virus are higher by 81% (OR = 1.81, p < 0.001, 95% CI: 1.31, 2.48) and 139% (OR = 2.39, p < .001, 95% CI: 1.66, 3.44), respectively. The odds that individuals with medium and high levels of trust in medical practitioners report washing their hands to mitigate the spread of the virus are 85% (OR = 1.85, p  = .002, 95% CI: 1.24, 2.72) and 134% (OR = 2.34, p < .001, 95% CI: 1.54, 3.52) higher compared to individuals who report no trust in medical practitioners.

Trust in both politicians and religious leaders was negatively associated with self-reported hand washing (Fig 4, Column 2). The odds that individuals with no trust in politicians report washing their hands to mitigate the spread of the virus are higher by 49% (OR = 1.49, p  = .02, 95% CI: 1.06, 2.09) compared to individuals who report high levels of trust in politicians. Similarly, the odds that individuals with no trust in religious leaders report washing their hands to mitigate the spread of the virus are higher by 65% (OR = 1.65, p < .01, 95% CI: 1.23, 2.20) compared to individuals who report high levels of trust in religious leaders.

### Social distancing behaviors

We found that individuals who reported greater confidence in both national health organizations and the WHO as well as greater trust in scientists also reported adopting social distancing behaviors to a greater extent (Fig 4, Column 3). The odds that individuals with medium and high levels of confidence in national health organizations have been adopting more social distancing behaviors to mitigate the spread of the virus are 26% (OR = 1.26, p < .01, 95% CI: 1.06, 1.49) and 48% (OR = 1.48, p < .001, 95% CI: 1.23, 1.78) higher compared to individuals with no trust in national health organizations. The odds that individuals with medium and high levels of confidence in the WHO have been adopting some social distancing behaviors (as opposed to none) to mitigate the spread of the virus are 23% (OR = 1.23, p < .01, 95% CI: 1.07, 1.40) and 29% (OR = 1.29, p  = .001, 95% CI: 1.12, 1.50) higher compared to individuals with no confidence in the WHO. Compared to individuals with no trust in scientists, the odds that individuals have been adopting more social distancing behaviors to mitigate the spread of the virus are 77% higher (OR = 1.77, p < .001, 95% CI: 1.42, 2.21) for those with medium levels of trust and 88% higher (OR = 1.88, p < .001, 95% CI: 1.48, 2.38) for those with high levels of trust in scientists. Confidence in local health organizations and trust in medical practitioners both had a null effect.

At the same time, individuals who reported more trust in politicians and religious leaders also reported less social distancing (Fig 4, Column 3). The odds that individuals with no trust in politicians have been adopting more social distancing behaviors to mitigate the spread of the virus are higher by 128% (OR = 2.28, p < .001, 95% CI: 1.92, 2.70) compared to individuals who report high levels of trust in politicians. Similarly, the odds that individuals with no trust in religious leaders have been adopting more social distancing behaviors to mitigate the spread of the virus are higher by 24% (OR = 1.24, p < .01, 95% CI: 1.08, 1.43) compared to individuals who report high levels of trust in religious leaders.

### Robustness checks

We tested the robustness of our findings in two ways (results not shown due to space constraints). First, we sequentially introduced the controls in our fully specified models. In the baseline model, we regressed only the trust and confidence measures on our three dependent variables. Next, we introduced our demographic controls, followed by COVID-19 related variables (risk perception, perceived policies), and finally personality traits. The statistically significant association between trust in politicians and religious leaders and mitigation behaviors is consistent across all specifications. Second, we estimated our specifications after sequentially deleting one country/territory from our sample at a time. Our findings indicate that the statistically significant association between trust in politicians and religious leaders and mitigation behaviors is not driven by any one country or territory within our sample. Both robustness checks focused on our findings regarding politicians and religious leaders because they are the only indicators of trust that are consistently significant across all three specifications of our dependent variable (hand-washing, mask wearing, and social distancing behaviors).

## Discussion

Although the promise of global vaccine distribution is slowly becoming a reality, progress remains uneven both across and within countries. Thus, in many contexts the best response locally and globally remains continued vigilance in adopting both personal and social mitigation behaviors. Moreover, just like the reach of the SARS-CoV-2 pandemic, our vigilance must be both local and global. Our research contributes to this effort by identifying common factors at the individual level that influence the adoption of personal and social mitigation behaviors across countries.

Specifically, we illuminate the influence of trust and confidence on individual compliance in three key ways. First, we explore whether there is a difference in the way trust and confidence in particular leaders and organizations affect individual compliance across multiple countries/territories at varying levels of development. Second, we explore whether this effect is consistent across different types of mitigation behaviors: personal and social distancing. Third, we consider the conditions under which trust might be negatively related to individual compliance. Taking into account several factors that have been identified as influencing individual compliance, including risk perception, socioeconomic status, and personality traits, our analyses show that trust and confidence remain significant. Across our sample, we find that trust in politicians and confidence in national health ministries are consistently associated with the adoption of both personal and social mitigation behaviors, but albeit in opposite directions.

We hypothesized that greater confidence in health organizations at three levels–local, national, and international–would be positively associated with the adoption of both personal and social mitigation behaviors. However, we find that this is only consistently the case when it comes to greater confidence in national health organizations, which is positively associated not only with increased mask wearing and handwashing but also with increased social distancing. Greater confidence in the WHO is also positively associated with increased adoption of social distancing behaviors, but it is not associated with the adoption of personal behaviors. Contrary to other studies [22], we do not find evidence that greater confidence in local health organizations is associated with the adoption of either personal or social distancing behaviors.

Our hypotheses for the relationship between trust and the adoption of mitigation behaviors differ by the type of domestic actor. We expected greater levels of trust in medical practitioners and scientists to be positively associated with the adoption of both personal and social mitigation behaviors. We found that greater trust in medical practitioners is positively associated only with hand washing and that greater trust in scientists is positively associated with both mask wearing and social distancing behaviors. Conversely, we expected greater levels of trust in politicians to be negatively associated with the adoption of both personal and social mitigation behaviors and greater levels of trust in religious leaders to be negatively associated with social distancing. Consistent with these expectations, we found that greater trust in politicians is negatively associated with mask wearing, hand washing, and social distancing and that greater trust in religious leaders is negatively associated with social distancing.

Our data has limitations as well as strengths. Because these data are cross-sectional, they can only provide a “snapshot” of the pandemic that will be most useful long-term when considered with the full spectrum of other studies conducted throughout the ongoing pandemic. The timing of our survey, however, is instructive. Conducted during the first wave of the SARS-CoV-2 pandemic (May-June 2020), it provides critical insights into how trust and confidence influence mitigation behaviors early on when preventative measures can be most effective in curtailing the spread of the virus. In addition, trust and confidence are essential to public health early in the pandemic trajectory when scientists have limited information on the disease and the need for compliance is great. While additional research on the role of confidence and trust is needed, these insights can help us to invest resources where they are likely to be most effective in curtailing future pandemics. The cross-national data from our survey is also limited geographically. We surveyed citizens in sixteen countries/territories across five world regions, and thus, cannot claim a truly global sample. Yet, with some important exceptions [38], most of the literature has been focused exclusively on high socio-demographic index (SDI) countries or countries within a single world region [13, 37]. The scope of our study thus improves our understanding of the factors that influence compliance by increasing the likelihood that our findings hold for citizens living in a variety of social and political contexts. While we cannot make inferences at the country/territory level, we can make them at the individual level across countries/territories in our sample.

In sum, our findings lend support to our contention that the role of trust and confidence in influencing the adoption of mitigation behaviors warrants greater attention. They indicate that trust and confidence should be considered as among the most important individual level characteristics driving compliance with recommended health behaviors during a pandemic. This, in turn, suggests the need to consider in which actors and institutions citizens place their trust and confidence when developing and delivering messaging about the adoption of mitigation behaviors. The content of the message, it seems, will be most effective when citizens across countries trust its source. Trusted sources, such as politicians and the national health ministry, should thus consider working closely together when determining and communicating recommended health behaviors to avoid contradicting one another.

## Supporting information

S1 Fig Confidence in health organization by country/territory. (TIF)

S2 Fig Trust in leaders by country/territory. (TIF)

S1 Table Summary statistics. (DOCX)

S2 Table Adjusted associations on adoption of COVID-19 mitigation behaviors. (DOCX)

S1 File (PDF)

S1 Data (CSV)
