# Supplementary figures and images for: Global adoption of personal and social mitigation behaviors during COVID-19: The role of trust & confidence
Source: PLoS One. 2021 Sep 8;16(9):e0256159. doi: 10.1371/journal.pone.0256159 (PMC8425551; doi:10.1371/journal.pone.0256159)

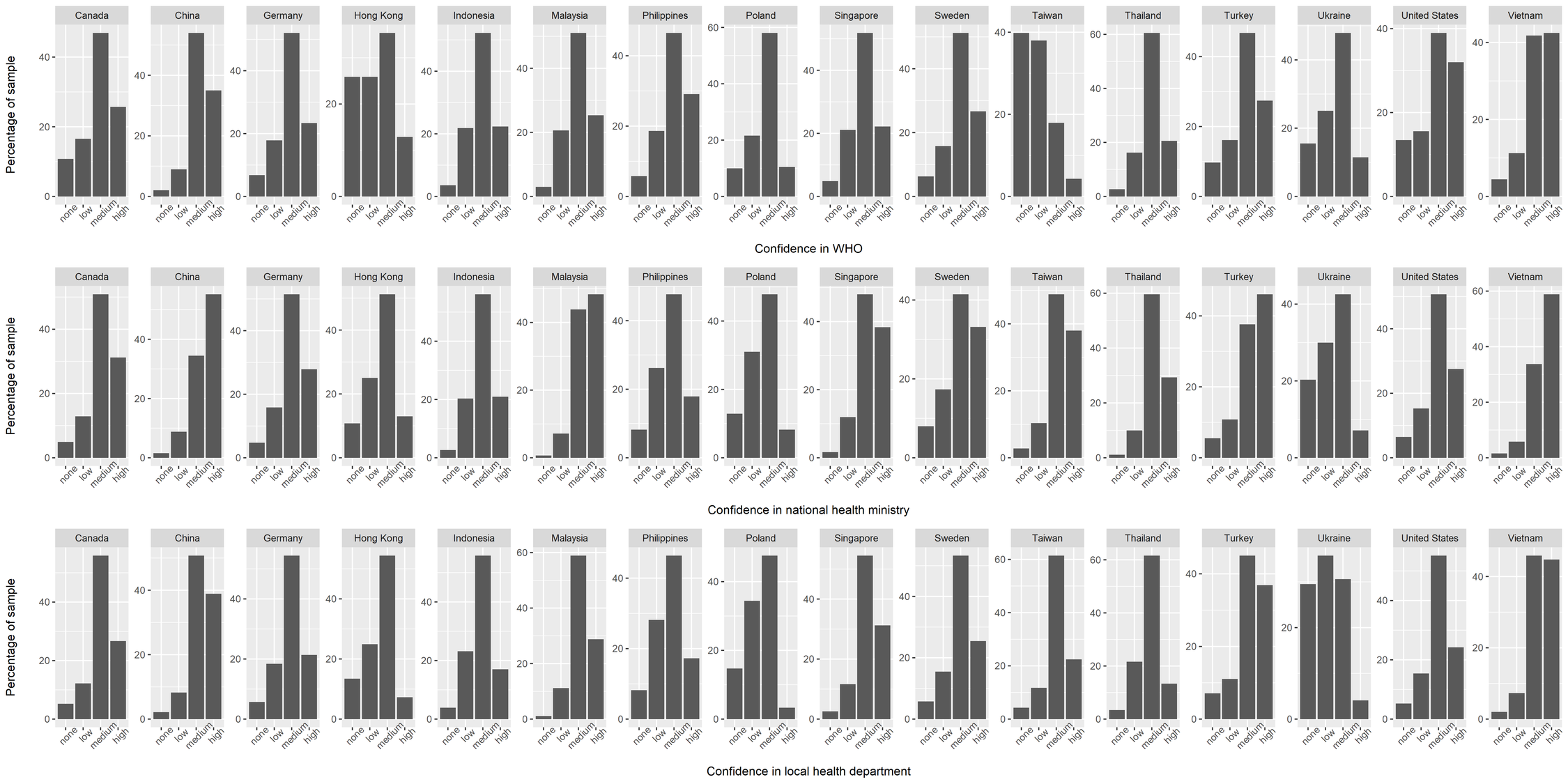

Supplement: S1 Fig — (TIF) [file pone.0256159.s001.tif]

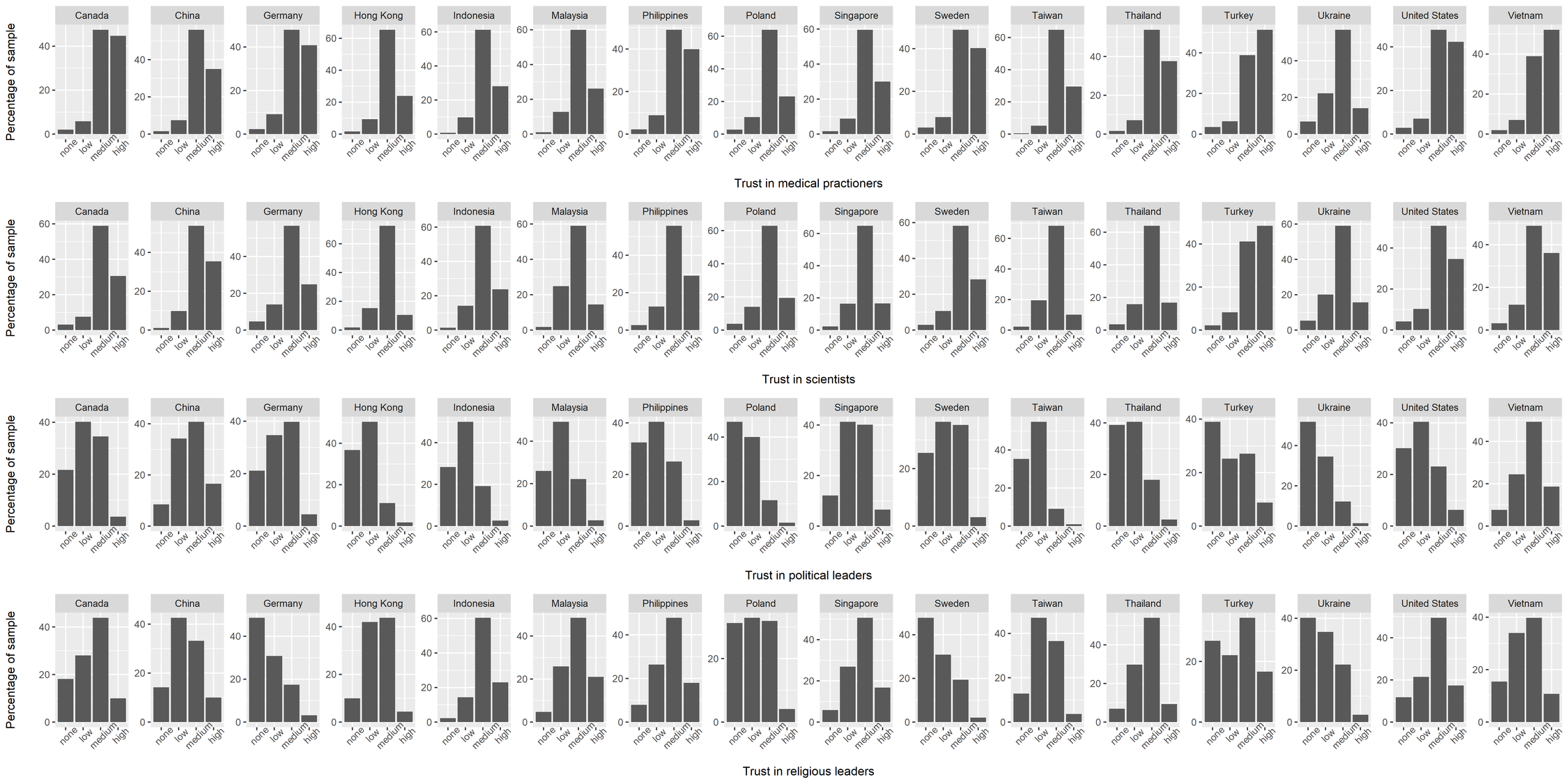

Supplement: S2 Fig — (TIF) [file pone.0256159.s002.tif]
