# Supplementary material for: Global adoption of personal and social mitigation behaviors during COVID-19: The role of trust & confidence
Source: PLoS One. 2021 Sep 8;16(9):e0256159. doi: 10.1371/journal.pone.0256159 (PMC8425551; doi:10.1371/journal.pone.0256159)
Supplement: S1 Table — (DOCX) [file pone.0256159.s003.docx]

**S1 Table: Summary Statistics**

| **Variable** | **N** | **Mean** | **S.D.** | **Median** | **Min** | **Max** |
| --- | --- | --- | --- | --- | --- | --- |
| Wearing masks | 16163 | 0.82 | 0.38 | 1 | 0 | 1 |
| Washing hands | 16163 | 0.92 | 0.27 | 1 | 0 | 1 |
| Social distancing index | 16163 | 1.77 | 1.07 | 2 | 0 | 3 |
| Trust – scientists | 15617 | 3.06 | 0.71 | 3 | 1 | 4 |
| Trust – politicians | 15653 | 2.08 | 0.87 | 2 | 1 | 4 |
| Trust – religious leaders | 15643 | 2.41 | 0.93 | 3 | 1 | 4 |
| Trust – medical practitioners | 15656 | 3.22 | 0.70 | 3 | 1 | 4 |
| Confidence – WHO | 15653 | 2.86 | 0.89 | 3 | 1 | 4 |
| Confidence – National health agency | 15638 | 3.03 | 0.84 | 3 | 1 | 4 |
| Confidence – Local health department | 15598 | 2.90 | 0.83 | 3 | 1 | 4 |
| Sex | 16567 | 0.49 | 0.50 | 0 | 0 | 1 |
| Age | 16604 | 2.17 | 0.87 | 2 | 1 | 4 |
| Education | 15524 | 3.65 | 1.21 | 4 | 1 | 6 |
| Socioeconomic status | 15249 | 3.57 | 1.24 | 3 | 1 | 5 |
| Infection concern – self | 15523 | 2.21 | 0.82 | 2 | 1 | 4 |
| Infection concern – family | 15470 | 2.62 | 0.92 | 3 | 1 | 4 |
| Perceived policies enforced – masks | 16046 | 0.66 | 0.48 | 1 | 0 | 1 |
| Perceived policies enforced – small groups only | 16150 | 0.68 | 0.47 | 1 | 0 | 1 |
| Perceived policies enforced – immediate household | 16026 | 0.39 | 0.49 | 0 | 0 | 1 |
| Big Five – extroversion | 15562 | 2.68 | 0.88 | 3 | 1 | 4 |
| Big Five – agreeableness | 15530 | 2.24 | 0.90 | 2 | 1 | 4 |
| Big Five – conscientiousness | 15541 | 3.21 | 0.71 | 3 | 1 | 4 |
| Big Five – neuroticism | 15535 | 2.39 | 0.90 | 2 | 1 | 4 |
| Big Five – openness to experience | 15522 | 3.11 | 0.76 | 3 | 1 | 4 |
