# Supplementary material for: Global adoption of personal and social mitigation behaviors during COVID-19: The role of trust & confidence
Source: PLoS One. 2021 Sep 8;16(9):e0256159. doi: 10.1371/journal.pone.0256159 (PMC8425551; doi:10.1371/journal.pone.0256159)
Supplement: S2 Table — (DOCX) [file pone.0256159.s004.docx]

**S2 Table: Adjusted associations on adoption of COVID-19 mitigation behaviors**

| **Variable** | **M1 -** *Mask wearing* | **M2 -** *Hand washing* | **M3 -** *Social distancing* |  |
| --- | --- | --- | --- | --- |
|  | *Coefficient (95% Confidence Interval)*  *(Standard Error) [p-value]* | | | |
| **Trust in** ^a^ |  | | | |
| Medical practitioners   - low - medium - high | -0.330 (-0.746, 0.077)  (0.210) [p = 0.116]  0.043 (-0.363, 0.437)  (0.204) [p = 0.834]  0.261 (-0.162, 0.674)  (0.213) [p = 0.220] | 0.105 (-0.298, 0.500)  (0.204) [p = 0.606]  0.614 (0.219, 0.999)  (0.199) [p = 0.002]  0.849 (0.430, 1.259)  (0.211) [p < 0.001] | -0.206 (-0.474, 0.061)  (0.137) [p = 0.131]  0.027 (-0.230, 0.284)  (0.131) [p = 0.836]  0.023 (-0.241, 0.287)  (0.135) [p = 0.862] |  |
| Religious leaders   - low - medium - high | 0.253 (0.073, 0.433)  (0.092) [p = 0.006]  0.010 (-0.174, 0.193)  (0.094) [p = 0.917]  -0.138 (-0.393, 0.118)  (0.130) [p = 0.288] | 0.136 (-0.068, 0.341)  (0.104) [p = 0.191]  -0.041 (-0.254, 0.171)  (0.108) [p = 0.708]  -0.498 (-0.789, -0.203)  (0.149) [p < 0.001] | -0.068 (-0.164, 0.029)  (0.049) [p = 0.168]  -0.130 (-0.230, -0.029)  (0.051) [p = 0.012]  -0.217 (-0.355, -0.079)  (0.071) [p = 0.002] |  |
| Politicians   - low - medium - high | -0.173 (-0.338, -0.008)  (0.084) [p = 0.040]  -0.548 (-0.740, -0.356)  (0.098) [p < 0.001]  -1.045 (-1.344, -0.742)  (0.154) [p < 0.001] | 0.121 (-0.069, 0.310)  (0.097) [p = 0.212]  -0.132 (-0.353, 0.089)  (0.113) [p = 0.242]  -0.400 (-0.735, -0.057)  (0.173) [p = 0.021] | -0.088 (-0.171, -0.006)  (0.042) [p = 0.037]  -0.350 (-0.450, -0.251)  (0.051) [p < 0.001]  -0.822 (-0.993, -0.651)  (0.087) [p < 0.001] |  |
| Scientists   - low - medium - high | 0.008 (-0.366, 0.375)  (0.189) [p = 0.966]  0.335 (-0.033, 0.694)  (0.185) [p = 0.071]  0.397 (0.006, 0.780)  (0.197) [p = 0.044] | 0.087 (-0.301, 0.467)  (0.196) [p = 0.657]  0.135 (-0.248, 0.508)  (0.193) [p = 0.482]  0.221 (-0.196, 0.628)  (0.210) [p = 0.294] | 0.377 (0.146, 0.609)  (0.118) [p = 0.001]  0.569 (0.343, 0.794)  (0.115) [p < 0.001]  0.633 (0.398, 0.869)  (0.120) [p < 0.001] |  |
| **Confidence in** ^b^ |  |  |  |  |
| WHO   - low - medium - high | -0.045 (-0.290, 0.197)  (0.124) [p = 0.717]  0.030 (-0.215, 0.272)  (0.124) [p = 0.807]  0.086 (-0.196, 0.366)  (0.143) [p = 0.549] | -0.231 (-0.499, 0.033)  (0.136) [p = 0.089]  -0.124 (-0.399, 0.146)  (0.139) [p = 0.371]  0.064 (-0.259, 0.386)  (0.165) [p = 0.696] | 0.157 (0.021, 0.293)  (0.069) [p = 0.024]  0.205 (0.071, 0.340)  (0.069) [p = 0.003]  0.258 (0.109, 0.408)  (0.076) [p = 0.001] |  |
| National health agency   - low - medium - high | 0.384 (0.106, 0.660)  (0.141) [p = 0.006]  0.614 (0.321, 0.904)  (0.149) [p < 0.001]  0.780 (0.451, 1.107)  (0.167) [p < 0.001] | 0.379 (0.077, 0.679)  (0.153) [p = 0.013]  0.591 (0.268, 0.909)  (0.163) [p < 0.001]  0.873 (0.507, 1.236)  (0.186) [p < 0.001] | 0.160 (-0.008, 0.329)  (0.086) [p = 0.062]  0.230 (0.059, 0.401)  (0.087) [p = 0.009]  0.391 (0.206, 0.577)  (0.095) [p < 0.001] |  |
| Local health department   - low - medium - high | -0.080 (-0.342, 0.179)  (0.133) [p = 0.549]  0.115 (-0.155, 0.382)  (0.137) [p = 0.400]  0.024 (-0.287, 0.333)  (0.158) [p = 0.878] | -0.068 (-0.358, 0.217)  (0.147) [p = 0.643]  0.038 (-0.263, 0.333)  (0.152) [p = 0.802]  0.009 (-0.343, 0.359)  (0.179) [p = 0.959] | -0.099 (-0.250, 0.052)  (0.077) [p = 0.201]  0.033 (-0.119, 0.185)  (0.078) [p = 0.670]  0.170 (-0.002, 0.341)  (0.088) [p = 0.053] |  |
| Socioeconomic status ^c^   - low - medium - high - highest | 0.242 (-0.039, 0.520)  (0.143) [p = 0.090]  0.421 (0.165, 0.672)  (0.129) [p = 0.001]  0.528 (0.245, 0.807)  (0.143) [p < 0.001]  0.535 (0.273, 0.793)  (0.133) [p < 0.001] | 0.271 (-0.040, 0.578)  (0.157) [p = 0.085]  0.489 (0.206, 0.764)  (0.142) [p = 0.001]  0.355 (0.047, 0.658)  (0.156) [p = 0.022]  0.637 (0.347, 0.919)  (0.146) [p < 0.001] | 0.105 (-0.051, 0.261)  (0.080) [p = 0.188]  0.135 (-0.005, 0.274)  (0.071) [p = 0.058]  0.227 (0.075, 0.380)  (0.078) [p = 0.004]  0.350 (0.206, 0.495)  (0.074) [p < 0.001] |  |
| Female (reference: male) | 0.048 (-0.071, 0.168)  (0.061) [p = 0.430] | 0.215 (0.076, 0.354)  (0.071) [p = 0.003] | 0.225 (0.162, 0.288)  (0.032) [p < 0.001] |  |
| Infection concern – self ^d^   - somewhat - very much - extremely | 0.445 (0.261, 0.628)  (0.094) [p < 0.001]  0.618 (0.377, 0.859)  (0.123) [p < 0.001]  0.707 (0.375, 1.044)  (0.170) [p < 0.001] | 0.295 (0.095, 0.491)  (0.101) [p = 0.004]  0.273 (-0.005, 0.552)  (0.142) [p = 0.055]  0.309 (-0.095, 0.723)  (0.209) [p = 0.139] | 0.224 (0.120, 0.329)  (0.054) [p < 0.001]  0.319 (0.186, 0.451)  (0.068) [p < 0. 001]  0.306 (0.136, 0.476)  (0.087) [p < 0. 001] |  |
| Infection concern – family ^e^   - somewhat - very much - extremely | 0.224 (0.009, 0.438)  (0.109) [p = 0.040]  0.381 (0.139, 0.622)  (0.123) [p = 0.002]  0.603 (0.320, 0.887)  (0.145) [p < 0.001] | 0.488 (0.267, 0.708)  (0.112) [p < 0.001]  0.847 (0.585, 1.110)  (0.134) [p < 0.001]  1.152 (0.826, 1.484)  (0.168) [p < 0.001] | 0.435 (0.305, 0.565)  (0.067) [p < 0. 001]  0.696 (0.553, 0.839)  (0.073) [p < 0. 001]  0.913 (0.752, 1.073)  (0.082) [p < 0. 001] |  |
| Education ^f^   - High school - Some college - 4-year college - Graduate/Professional - Doctorate | 0.042 (-0.304, 0.379)  (0.174) [p = 0.809]  0.031 (-0.322, 0.375)  (0.178) [p = 0.863]  0.361 (0.015, 0.698)  (0.174) [p = 0.038]  0.125 (-0.228, 0.469)  (0.178) [p = 0.483]  0.290 (-0.144, 0.722)  (0.221) [p = 0.189] | 0.462 (0.105, 0.804)  (0.178) [p = 0.010]  0.369 (0.002, 0.723)  (0.184) [p = 0.044]  0.278 (-0.077, 0.619)  (0.177) [p = 0.116]  0.395 (0.027, 0.750)  (0.184) [p = 0.032]  0.305 (-0.152, 0.761)  (0.233) [p = 0.189] | 0.109 (-0.091, 0.308)  (0.102) [p = 0.286]  0.148 (-0.056, 0.353)  (0.104) [p = 0.154]  0.223 (0.024, 0.422)  (0.101) [p = 0.028]  0.146 (-0.058, 0.349)  (0.104) [p = 0.160]  0.250 (0.005, 0.495)  (0.125) [p = 0.045] |  |
| Age ^g^   - 30 – 49 - 50 – 69 - 70 + | -0.221 (-0.384, -0.059)  (0.083) [p = 0.008]  -0.068 (-0.243, 0.105)  (0.089) [p = 0.441]  -0.034 (-0.290, 0.224)  (0.131) [p = 0.793] | -0.088 (-0.276, 0.098)  (0.095) [p = 0.358]  0.009 (-0.194, 0.209)  (0.103) [p = 0.934]  -0.121 (-0.420, 0.184)  (0.154) [p = 0.432] | 0.187 (0.106, 0.268)  (0.041) [p < 0. 001]  0.504 (0.415, 0.593)  (0.046) [p < 0. 001]  0.853 (0.701, 1.004)  (0.077) [p < 0. 001] |  |
| Perceived policies enforced   - Masks - Only small groups - Only immediate family | 0.950 (0.816, 1.084)  (0.068) [p < 0.001]  0.152 (0.006, 0.298)  (0.074) [p = 0.042]  -0.206 (-0.349, -0.064)  (0.073) [p = 0.005] | 0.320 (0.162, 0.478)  (0.081) [p < 0.001]  0.270 (0.105, 0.434)  (0.084) [p = 0.001]  -0.049 (-0.213, 0.117)  (0.084) [p = 0.564] | 0.228 (0.151, 0.306)  (0.040) [p < 0.001]  0.217 (0.139, 0.296)  (0.040) [p < 0.001]  -0.074 (-0.149, 0.002)  (0.039) [p = 0.056] |  |
| **Big Five** ^h^ |  |  |  |  |
| Extroversion   - Disagree a little - Agree a little - Agree strongly | -0.134 (-0.340, 0.070)  (0.105) [p = 0.199]  0.008 (-0.196, 0.209)  (0.103) [p = 0.941]  -0.035 (-0.289, 0.218)  (0.129) [p = 0.785] | 0.131 (-0.098, 0.358)  (0.116) [p = 0.259]  0.217 (-0.010, 0.441)  (0.115) [p = 0.059]  0.134 (-0.156, 0.424)  (0.148) [p = 0.366] | -0.019 (-0.133, 0.095)  (0.058) [p = 0.739]  -0.036 (-0.147, 0.075)  (0.057) [p = 0.527]  -0.016 (-0.150, 0.118)  (0.069) [p = 0.821] |  |
| Agreeableness   - Disagree a little - Agree a little - Agree strongly | -0.146 (-0.320, 0.026)  (0.088) [p = 0.098]  -0.254 (-0.437, -0.073)  (0.093) [p = 0.006]  -0.581 (-0.843, -0.317)  (0.134) [p < 0.001] | 0.044 (-0.165, 0.250)  (0.106) [p = 0.681]  -0.264 (-0.478, -0.052)  (0.109) [p = 0.015]  -0.674 (-0.962, -0.382)  (0.148) [p < 0.001] | -0.119 (-0.207, -0.031)  (0.045) [p = 0.008]  -0.149 (-0.243, -0.054)  (0.048) [p = 0.002]  -0.227 (-0.371, -0.082)  (0.074) [p = 0.002] |  |
| Conscientiousness   - Disagree a little - Agree a little - Agree strongly | -0.003 (-0.406, 0.390)  (0.203) [p = 0.987]  0.149 (-0.231, 0.518)  (0.191) [p = 0.437]  0.230 (-0.154, 0.604)  (0.193) [p = 0.233] | -0.298 (-0.741, 0.128)  (0.222) [p = 0.179]  0.010 (-0.413, 0.413)  (0.211) [p = 0.961]  0.073 (-0.357, 0.482)  (0.214) [p = 0.734] | 0.084 (-0.160, 0.328)  (0.125) [p = 0.500]  0.286 (0.056, 0.516)  (0.117) [p = 0.015]  0.497 (0.265, 0.730)  (0.119) [p < 0.001] |  |
| Neuroticism   - Disagree a little - Agree a little - Agree strongly | 0.061 (-0.121, 0.242)  (0.092) [p = 0.509]  0.043 (-0.149, 0.234)  (0.098) [p = 0.659]  -0.119 (-0.362, 0.125)  (0.124) [p = 0.338] | 0.280 (0.073, 0.485)  (0.105) [p = 0.008]  0.086 (-0.130, 0.299)  (0.110) [p = 0.435]  0.031 (-0.245, 0.311)  (0.142) [p = 0.825] | -0.026 (-0.119, 0.068)  (0.048) [p = 0.595]  0.003 (-0.096, 0.010)  (0.051) [p = 0.956]  -0.120 (-0.251, 0.011)  (0.067) [p = 0.073] |  |
| Openness to experience   - Disagree a little - Agree a little - Agree strongly | 0.309 (-0.045, 0.654)  (0.178) [p = 0.083]  0.410 (0.069, 0.743)  (0.172) [p = 0.017]  0.472 (0.116, 0.819)  (0.179) [p = 0.008] | 0.249 (-0.106, 0.595)  (0.179) [p = 0.164]  0.443 (0.098, 0.778)  (0.173) [p = 0.011]  0.589 (0.221, 0.947)  (0.185) [p = 0.002] | 0.244 (0.034, 0.455)  (0.107) [p = 0.023]  0.377 (0.173, 0.581)  (0.104) [p < 0.001]  0.453 (0.243, 0.664)  (0.107) [p < 0.001] |  |
| **Nagelkerke R^2^** | 0.500 | 0.172 | 0.174 |  |
| **Country Fixed Effects** | Yes | Yes | Yes |  |
| **Observations** | 13,996 | 13,996 | 13,996 |  |

Notes: Coefficients, standard errors, p-values, and 95% confidence intervals are retrieved from binomial logistic regressions for Models 1 and 2, and an ordered logistic regression for Model 3.

a – Reference group: no trust

b – Reference group: no confidence

c – Reference group: lowest socioeconomic grouping

d – Reference group: no concern of themselves being infected

e – Reference group: no concern of their loved ones being infected

f – Reference group: Less than high school education

g – Reference group: 18-29 years of age

h – Reference group: disagree strongly that attribute applies to respondent
