# Supplementary material for: Global adoption of personal and social mitigation behaviors during COVID-19: The role of trust & confidence
Source: PLoS One. 2021 Sep 8;16(9):e0256159. doi: 10.1371/journal.pone.0256159 (PMC8425551; doi:10.1371/journal.pone.0256159)
Supplement: S1 File — (PDF) [file pone.0256159.s005.pdf]

CoVid-19 - Extended - International Revised

Survey Flow

Start of Block: Informed Consent

Q1 **Understanding Decision making About Coronavirus**
The purpose of this study is to investigate how people make decisions about coping with the current Coronavirus (COVID-19) pandemic.
We expect the entire survey to take 15-20 minutes to complete. You may **save your responses and come back** to the survey within one week after you have started it.

Compensation You will be compensated the amount you agreed upon before you entered into the survey.
Confidentiality
Your responses will not be linked to your identity and cannot be identified in any reports on this survey. We recommend that you complete it in a private space where nobody else can see your responses.

Participation & Consent
Your participation in this study is voluntary. You may refuse to answer any survey question without affecting your study compensation.

By checking the box below, you have indicated that you have read and understood the above information, and that you agree to participate in this study.

Contact Information
Questions, comments, or concerns about this research study can be directed to Twila Tardif, email: twila@umich.edu.
If you have questions about your rights as a research participant, or wish to obtain information, ask questions or discuss any concerns about this study with someone other than the researcher(s), please contact the University of Michigan Health Sciences and Behavioral Sciences Institutional Review Board (IRB-HSBS) , 2800 Plymouth Rd., Building 520, Room 1169 , Ann Arbor, MI 48109-2800 , Phone: 734-936-0933 , Fax: 734-936-1852 , Email:

irbhsbs@umich.edu. eResearch ID: HUM00168677

- ◯ Yes, I understand the above information and agree to participate in the study. (1)
- ◯ No, I do not wish to participate in the study (2)

Skip To: End of Block If Q1 = 2

End of Block: Informed Consent

Start of Block: Location

Q2 In which country do you currently reside?

▼ United States of America (1) ... Viet Nam (192)

Display This Question:

If Q2 != 36

Q3 Please answer the following question before continuing to the Survey

Q4 What is your postal (zip) code?

____

Display This Question:

If Q2 = 143

Or Q2 = 16

Q5 What region do you live in? (Russia and Belarus)

▼ Click to write Choice 1 (4) ... Click to write Choice 86 (89)

Display This Question:

If Q2 = 143

Or Q2 = 16

Q6 What is the population of your residence? (Russia and Belarus)

- ○ More than 1 million (1)
- ○ 500-1 million (4)
- ○ 100,000-500,000 (5)
- ○ Less than 100,000 (6)

Page Break

Display This Question:

If Q2 = 143

Or Q2 = 16

Q7 Where do you live? (Russia and Belarus)

◯ Rural area (1)

◯ City (4)

End of Block: Location

Start of Block: Have you ever lived in another country?

Q8 Have you ever lived in any other country for six months or more?

◯ No (2)

◯ Yes (1)

Display This Question:

If Q8 = 1

Q9 In which countries have you lived for 6 months or more? (Select all that apply - if using a computer hold down the CTRL or Command key)

- ☐ United States of America (1)
- ☐ Albania (2)
- ☐ Algeria (3)
- ☐ Argentina (7)
- ☐ Armenia (8)
- ☐ Australia (9)
- ☐ Austria (10)
- ☐ Azerbaijan (11)
- ☐ Bahamas (12)
- ☐ Bangladesh (14)
- ☐ Barbados (15)
- ☐ Belarus (16)
- ☐ Belgium (17)
- ☐ Belize (18)
- ☐ Bolivia (21)
- ☐ Bosnia and Herzegovina (22)
- ☐ Botswana (1359)
- ☐ Brazil (24)

- [ ] Bulgaria (26)
- [ ] Burundi (28)
- [ ] Cambodia (29)
- [ ] Cameroon (30)
- [ ] Canada (31)
- [ ] Central African Republic (33)
- [ ] Chad (34)
- [ ] Chile (35)
- [ ] China (36)
- [ ] Colombia (37)
- [ ] Congo, Republic of the... (39)
- [ ] Costa Rica (40)
- [ ] Côte d'Ivoire (41)
- [ ] Croatia (42)
- [ ] Cuba (43)
- [ ] Cyprus (44)
- [ ] Czech Republic (45)
- [ ] Democratic People's Republic of Korea (46)

- [ ] Democratic Republic of the Congo (47)
- [ ] Denmark (48)
- [ ] Dominica (50)
- [ ] Dominican Republic (51)
- [ ] Ecuador (52)
- [ ] Egypt (53)
- [ ] El Salvador (54)
- [ ] Equatorial Guinea (55)
- [ ] Eritrea (56)
- [ ] Estonia (57)
- [ ] Ethiopia (58)
- [ ] Fiji (59)
- [ ] Finland (60)
- [ ] France (61)
- [ ] Gambia (63)
- [ ] Georgia (64)
- [ ] Germany (65)
- [ ] Ghana (66)

- [ ] Greece (67)
- [ ] Grenada (68)
- [ ] Guatemala (69)
- [ ] Guinea (70)
- [ ] Guyana (72)
- [ ] Haiti (73)
- [ ] Honduras (74)
- [ ] Hong Kong (S.A.R.) (75)
- [ ] Hungary (76)
- [ ] Iceland (77)
- [ ] India (78)
- [ ] Indonesia (79)
- [ ] Iran, Islamic Republic of... (80)
- [ ] Iraq (81)
- [ ] Ireland (82)
- [ ] Israel (83)
- [ ] Italy (84)
- [ ] Jamaica (85)

- [ ] Japan (86)
- [ ] Jordan (87)
- [ ] Kazakhstan (88)
- [ ] Kenya (89)
- [ ] Kuwait (91)
- [ ] Kyrgyzstan (92)
- [ ] Lao People's Democratic Republic (93)
- [ ] Latvia (94)
- [ ] Lebanon (95)
- [ ] Lesotho (96)
- [ ] Liberia (97)
- [ ] Libyan Arab Jamahiriya (98)
- [ ] Liechtenstein (99)
- [ ] Lithuania (100)
- [ ] Luxembourg (101)
- [ ] Macao (S.A.R.) (1358)
- [ ] Madagascar (102)
- [ ] Malawi (103)

- [ ] Malaysia (104)
- [ ] Maldives (105)
- [ ] Mali (106)
- [ ] Malta (107)
- [ ] Mauritania (109)
- [ ] Mauritius (110)
- [ ] Mexico (111)
- [ ] Mongolia (114)
- [ ] Montenegro (115)
- [ ] Morocco (116)
- [ ] Mozambique (117)
- [ ] Myanmar (118)
- [ ] Namibia (119)
- [ ] Nepal (121)
- [ ] Netherlands (122)
- [ ] New Zealand (123)
- [ ] Nicaragua (124)
- [ ] Niger (125)

- [ ] Nigeria (126)
- [ ] Norway (128)
- [ ] Oman (129)
- [ ] Pakistan (130)
- [ ] Panama (132)
- [ ] Papua New Guinea (133)
- [ ] Paraguay (134)
- [ ] Peru (135)
- [ ] Philippines (136)
- [ ] Poland (137)
- [ ] Portugal (138)
- [ ] Qatar (139)
- [ ] Republic of Korea (140)
- [ ] Republic of Moldova (141)
- [ ] Romania (142)
- [ ] Russian Federation (143)
- [ ] Rwanda (144)
- [ ] Samoa (148)

- [ ] Saudi Arabia (151)
- [ ] Senegal (152)
- [ ] Serbia (153)
- [ ] Sierra Leone (155)
- [ ] Singapore (156)
- [ ] Slovakia (157)
- [ ] Slovenia (158)
- [ ] Solomon Islands (159)
- [ ] Somalia (160)
- [ ] South Africa (161)
- [ ] Spain (163)
- [ ] Sri Lanka (164)
- [ ] Sudan (165)
- [ ] Suriname (166)
- [ ] Eswatini (167)
- [ ] Sweden (168)
- [ ] Switzerland (169)
- [ ] Syrian Arab Republic (170)

- [ ] Taiwan (173)
- [ ] Tajikistan (171)
- [ ] Thailand (172)
- [ ] Timor-Leste (174)
- [ ] Togo (175)
- [ ] Tonga (176)
- [ ] Trinidad and Tobago (177)
- [ ] Tunisia (178)
- [ ] Turkey (179)
- [ ] Turkmenistan (180)
- [ ] Uganda (182)
- [ ] Ukraine (183)
- [ ] United Arab Emirates (184)
- [ ] United Kingdom of Great Britain and Northern Ireland (185)
- [ ] United Republic of Tanzania (186)
- [ ] Uruguay (188)
- [ ] Uzbekistan (189)
- [ ] Vanuatu (190)

☐ Venezuela, Bolivarian Republic of... (191)

☐ Viet Nam (192)

☐ Yemen (193)

☐ Zambia (580)

☐ Zimbabwe (1357)

End of Block: Have you ever lived in another country?

Start of Block: Age, gender and living status

*

Q79 What is your year of birth?

____

Skip To: End of Block If Condition: What is your year of birth? Is Greater Than 2002. Skip To: End of Block.

Skip To: End of Block If Condition: What is your year of birth? Is Less Than 1900. Skip To: End of Block.

Q10 What is your sex?

▼ Male (1) ... Other or prefer not to answer (3)

Q11 Who do you live with? (Select all that apply.)

	Before COVID pandemic (1)	During COVID pandemic (2)
I live alone (7)	☐	☐
I live with children (1)	☐	☐
I live with my parents (2)	☐	☐
I live with other family members (e.g., aunt, uncle, siblings) (3)	☐	☐
I live with a spouse or common-law partner (4)	☐	☐
I live with non-family members (5)	☐	☐
Prefer not to say (6)	☐	☐

Display This Question:

If Q11 = 1 [2]

Q12 How many children, aged 25 or below, are currently living in your household?

- ○ 1 (1)
- ○ 2 (2)
- ○ 3 (3)
- ○ 4 (4)
- ○ 5 (5)
- ○ 6 or more (6)

Display This Question:

If Q11 = 1 [2]

Q13 Which of the following describes the children in your household?(Please select all that apply)

☐ Too young for school (1)

☐ In elementary school (2)

☐ In middle school (3)

☐ In high school (4)

☐ In post-secondary education (e.g., college, university, CEGEP) (5)

☐ No longer in school (6)

☐ ⊗Prefer not to say (7)

Display This Question:

If Q11 = 1 [2]

Q14 What are your current concerns about your children during the COVID-19 pandemic?

- [ ] They might become sick with the coronavirus (1)
- [ ] Other physical health concerns (2)
- [ ] They might infect me with coronavirus (3)
- [ ] Their mental health (4)
- [ ] Their ability to make academic progress (5)
- [ ] Social isolation (6)
- [ ] Missing out on sports (7)
- [ ] ⊗None (9)
- [ ] Other (8) ____

End of Block: Age, gender and living status

Start of Block: Coronavirus Government Restrictions

Q15 The next set of questions ask about **actions** that you and your local government may or may not be taking to minimize the spread of COVID-19. Please answer **to the best of your knowledge** at this time.

Q16 What action has **your government (local or national)** enacted to restrict the spread of COVID-19?

	Never in force (1)	Previously in force but not now (2)	Currently in force (3)	Not sure (4)
Restricted gatherings to small number of people (2)	○	○	○	○
Restricted gatherings to people in immediate household (3)	○	○	○	○
"Work from home" for all but essential personnel (4)	○	○	○	○
Closed schools (5)	○	○	○	○
Closed places of worship (6)	○	○	○	○
Closed gyms or exercise facilities (26)	○	○	○	○
Closed restaurants/bars (8)	○	○	○	○
Restaurants/bars take-out only (20)	○	○	○	○
Seating restrictions inside restaurants (21)	○	○	○	○
Limited hours for grocery stores and pharmacies (9)	○	○	○	○
Limited number of people inside stores (10)	○	○	○	○
Arrows/marks on public floors to restrict foot traffic (22)	○	○	○	○
Closed playgrounds and parks (11)	○	○	○	○

Required face masks in all public places (12)	○	○	○	○
Explicit permission needed to leave the home (16)	○	○	○	○
Limited number of people leaving your home (7)	○	○	○	○
Provide a route indicating where you will go (23)	○	○	○	○
Mandatory testing for COVID-19 (27)	○	○	○	○
Curfews (13)	○	○	○	○
Restricted days for driving (24)	○	○	○	○
Restricted travel in/out of my city (18)	○	○	○	○
14-days mandatory isolation for new arrivals to my city/country (19)	○	○	○	○

Page Break

Q17 Which of the following **steps have you taken** to try to minimize the spread of the COVID-19 in your community? (Select all that apply.)

- ▢ Washing your hands more often (1)
- ▢ Using alcohol-based hand sanitizer more often (12)
- ▢ Wearing a face mask (13)
- ▢ Avoiding social events (e.g., parties, family gatherings) (14)
- ▢ Avoiding public transit (15)
- ▢ Avoiding any enclosed spaces outside your home (16)
- ▢ Touching your face less (17)
- ▢ Shopping for groceries less often (18)
- ▢ Cooking at home more often (19)
- ▢ Staying home from work (20)
- ▢ Purchasing extra supplies or food (21)
- ▢ Not going outside your home for walks or exercise (22)
- ▢ Avoiding physical contact when greeting friends and family members (25)
- ▢ Cleaning my living space more often (28)
- ▢ Cleaning groceries and all things that get delivered to my living space (29)
- ▢ I am not doing anything differently (24)

Page Break

End of Block: Coronavirus Government Restrictions

Start of Block: Coronavirus - Social Distancing

Q18 To what extent are **you** currently "social distancing" (i.e., avoiding contact and maintaining at least 6 feet/2 meters between you and individuals outside your household)?

▼ None of the time (7) ... All of the time (10)

Q19 To what extent do you think **others in your community** are currently "social distancing" (i.e., avoiding contact and maintaining at least 6 feet/2 meters between you and individuals outside your household)?

▼ None of the time (1) ... All of the time (4)

Page Break

Q20 Have you changed the way you celebrate any of the following events?

	No (1)	Somewhat (2)	A Lot (3)	Not Applicable (4)
Birthdays (11)	◯	◯	◯	◯
Q2 != 179<br>Cinco de Mayo (1)	◯	◯	◯	◯
Q2 != 179<br>Easter (2)	◯	◯	◯	◯
Q2 != 143<br>*And Q2 != 16*<br>Funerals (3)	◯	◯	◯	◯
Q2 != 183<br>May 1st (12)	◯	◯	◯	◯
Nouruz (9)	◯	◯	◯	◯
Q2 != 179<br>Passover (4)	◯	◯	◯	◯
Ramadan (6)	◯	◯	◯	◯
Q2 != 143<br>*And Q2 != 183*<br>*And Q2 != 16*<br>Songkran (7)	◯	◯	◯	◯
Q2 != 143<br>*And Q2 != 16*<br>*And Q2 != 183*<br>Qingming (8)	◯	◯	◯	◯
Graduation (13)	◯	◯	◯	◯
Valentine's Day (14)	◯	◯	◯	◯
Other (10)	◯	◯	◯	◯

Page Break

Display This Question:

If Q18 != 7

Q21 How long do you think you will be able to maintain your current level of social distancing?

▼ 1-2 days (1) ... 6 months or more (12)

Display This Question:

If Q18 != 7

Q22 How difficult is it for you to maintain your current level of social distancing?

- Not difficult (1)
- Somewhat difficult (4)
- Very difficult (5)
- Extremely difficult (6)

Q23 When do you think your community will be "safe" from the COVID-19 pandemic?

▼ It's safe now (1) ... 18 months or longer (8)

Page Break

Q24 What happens if you **violate** government guidelines on **social distancing**? (Select all that apply)

▢ Nothing (1)

▢ Warning (2)

▢ Small fine (no more than one day's salary) (7)

▢ Large fine (more than one day's salary) (3)

▢ Mandatory Detention (at home or elsewhere) (8)

▢ Jail/Prison time (5)

▢ Don't know (6)

End of Block: Coronavirus - Social Distancing

Start of Block: Coronavirus - Difficulties and Transitional Impact Scale (Svob et al, 2014)

Q25 The next set of questions ask to what extent the COVID-19 pandemic has affected aspects of your everyday life. Some people have experienced a great deal of difficulty and change, whereas other people have not. We are only interested in how much the COVID-19 pandemic has affected **your daily life and plans.**

Q26 Have you experienced any difficulties during the COVID-19 crisis? (Select all that apply)

- ▢ Loneliness (1)
- ▢ Cancellation of travel (2)
- ▢ Reduced income (3)
- ▢ Relocation of where I live (4)
- ▢ Increased anxiety (5)
- ▢ Obtaining groceries (6)
- ▢ Accessing healthcare (7)
- ▢ Accessing exercise facilities (16)
- ▢ Boredom (8)
- ▢ Loss of job (9)
- ▢ Family tensions and stress (15)
- ▢ Childcare (10)
- ▢ Obtaining medication (11)
- ▢ Transportation (12)
- ▢ I have not experienced any difficulties during the COVID-19 crisis (14)

Page Break

Q27 The Coronavirus (COVID-19) crisis has changed or impacted...

	Strongly disagree (14)	Somewhat disagree (15)	Somewhat agree (17)	Strongly agree (18)
My plans for the future. (1)	○	○	○	○
My material circumstances. (3)	○	○	○	○
The activities I engage in. (4)	○	○	○	○
The people I spend time with. (5)	○	○	○	○
Where I live. (6)	○	○	○	○
My attitudes about life. (7)	○	○	○	○
The way I think about things. (10)	○	○	○	○
My mental health. (12)	○	○	○	○

Page Break

Q28 The frequency and/or intensity of arguments **my partner** and I have experienced since the COVID-19 crisis began has:

▼ decreased (1) ... Not relevant (5)

Q29 The frequency and/or intensity of arguments my **other family members** and I have experienced since the COVID-19 crisis began has:

▼ decreased (1) ... Not relevant (5)

End of Block: Coronavirus - Difficulties and Transitional Impact Scale (Svob et al, 2014)

Start of Block: Coronavirus sources of information and Conspiracy Theories

Q30 The next set of questions ask about where you learn information about COVID-19 and your **current understanding** of how it is spread.

Page Break

Q31 Compared to before the COVID pandemic, how has my **news media** consumption **changed**?

	Decreased (1)	Stayed Same (4)	Increased (5)
At the beginning of the pandemic (5)	○	○	○
Currently (6)	○	○	○

Q32 About how much **time** do you **currently** spend consuming news media coverage of COVID-19 **each day**?

▼ 0-30 minutes (0.5 hours or less) (1) ... more than 240 minutes (more than 4 hours) (5)

Q33 What **sources** do you use to obtain reliable information about COVID-19? (Select all that apply)

- ▢ Friends or family members (1)
- ▢ Social Media (e.g., Twitter, WeChat, Facebook, Whatsapp) (2)
- ▢ National TV or radio news (3)
- ▢ National Newspapers and Magazines (including online versions) (5)
- ▢ International news media (11)
- ▢ Briefings by political leaders (7)
- ▢ National or international health department websites (e,g., WHO or CDC) (8)
- ▢ Local health department websites (10)
- ▢ Scientific articles (9)
- ▢ My own doctor or healthcare provider (12)
- ▢ Religious leader(s) (13)
- ▢ Other (specify) (6) ____

Page Break

Q34 How much do you **agree** with the following statements about COVID-19 (Coronavirus)?

	Disagree (7)	Agree (6)	Don't Know (8)
It only affects older people. (1)	○	○	○
It has a higher death rate for people over 70. (2)	○	○	○
Children and teenagers are immune from it. (15)	○	○	○
It is no different from seasonal flu. (4)	○	○	○
My government is over-reporting the number of people who have died from it. (19)	○	○	○
My government is under-reporting the number of people who have died from it. (6)	○	○	○
It will kill more people than the flu this year. (8)	○	○	○
The World Health Organization (WHO) has exaggerated the danger of COVID-19. (18)	○	○	○
A vaccine for it is likely to be available to me within the next six months. (9)	○	○	○
It originated in a laboratory doing research on biological warfare. (5)	○	○	○
It originated in animals. (10)	○	○	○
I could catch it if someone infected with it coughs on me. (13)	○	○	○
It will stay in the air for several hours after someone coughs or sneezes. (14)	○	○	○

It's important to avoid touching any surface that people outside my household may have touched. (16)	○	○	○
International news is more reliable than my national news for information about it. (17)	○	○	○
The symptoms of it seem to be connected to 5G mobile network radiation. (11)	○	○	○

End of Block: Coronavirus sources of information and Conspiracy Theories

Start of Block: Past Month Feelings, Physical Symptoms, and Brief COPE - Coronavirus Adapted

Q35 The next set of questions ask about how you have been **feeling and coping** during the COVID-19 pandemic. Many people around the world have found the pandemic and the measures that have been taken to minimize its spread to be stressful. You may or may not feel this way, so we are interested only in how **you** are feeling at this time.

Page Break

Q36 How much have you been feeling the following emotions during the pandemic, relative to how much you experienced them before the pandemic?

	Less (5)	Same (6)	More (7)
Lonely (1)	○	○	○
Depressed (2)	○	○	○
Anxious (3)	○	○	○
Angry (18)	○	○	○
Happy (19)	○	○	○
Relaxed (20)	○	○	○
Focused (21)	○	○	○

Page Break

Q37 How much have you been doing the following actions during the pandemic, relative to how much you did them before the pandemic?

	Less (1)	Same (2)	More (3)
Working/doing schoolwork. (1)	○	○	○
Using alcohol or other drugs. (4)	○	○	○
Eating due to stress or boredom. (17)	○	○	○
Seeking emotional support from others. (5)	○	○	○
Helping others (7)	○	○	○
Feeling grateful (8)	○	○	○
Engaging in creative activities (9)	○	○	○
Journaling (10)	○	○	○
Sharing funny memes and videos. (11)	○	○	○
Sleeping (12)	○	○	○
Exercising. (13)	○	○	○
Praying or meditating. (14)	○	○	○
Watching movies/TV (16)	○	○	○
Spending time on social media. (18)	○	○	○
Reading/listening to the news. (19)	○	○	○
Avoiding the news. (20)	○	○	○
Playing games (21)	○	○	○
Connecting with family/friends (22)	○	○	○

End of Block: Past Month Feelings, Physical Symptoms, and Brief COPE - Coronavirus Adapted

Start of Block: Health

Q38 The next set of questions ask about your general health as well as whether you or anybody you know has been diagnosed with or suspected to have COVID-19.

Q39 In general, how is your health?

▼ Very good (1) ... Very Bad (5)

Q40 Which of the following **chronic** health conditions do you have? Check all that apply (even if controlled with medication)

- ☐ Heart disease (1)
- ☐ High blood pressure (12)
- ☐ Cancer (13)
- ☐ Diabetes (14)
- ☐ Asthma (15)
- ☐ Lung disease (16)
- ☐ Kidney or Liver disease (22)
- ☐ Immunocompromised (24)
- ☐ Obesity (23)
- ☐ Depression (17)
- ☐ Anxiety (18)
- ☐ None (20)
- ☐ Prefer not to answer (21)
- ☐ Other (Specify) (19) ____

Display This Question:

If Q2 != 16

And Q2 != 143

Q41 Are you **currently** (today) experiencing any of the following?

	No (1)	Some (2)	Extreme (3)
Problems walking (1)	○	○	○
Problems washing or dressing (2)	○	○	○
Problems with daily activities (e.g., school, work, housework, family, or leisure activities) (3)	○	○	○
Pain or discomfort (4)	○	○	○
Anxiety/Depression (5)	○	○	○

Q42 Which of these physical symptoms have you experienced in the **past (1) month**?

	No (2)	Somewhat (8)	Often (3)
Headaches (1)	○	○	○
Sick to my stomach (2)	○	○	○
Heart beating fast (3)	○	○	○
Hard to breathe (4)	○	○	○
Feel so restless I can't sit still (6)	○	○	○
Muscles feel tense or sore (9)	○	○	○
Problems sleeping (16)	○	○	○

Q43 In the **past 3 months**, have you had any of the following symptoms?

- ☐ Fever, chills, or sweating (1)
- ☐ Sore throat (2)
- ☐ Cough unrelated to allergies or chronic condition (3)
- ☐ Body aches (4)
- ☐ Persistent exhaustion (5)
- ☐ Difficulty breathing (6)
- ☐ Vomiting or diarrhea (7)
- ☐ Anosmia (loss of smell) (8)
- ☐ No, I have not had any of the above symptoms (10)
- ☐ Other (please describe) (9) ____

Page Break

End of Block: Health

Start of Block: Russian version EQ5D

Display This Question:

If Q2 = 143

Or Q2 = 16

Q141 Check ONE box in each question below. Indicate the answers that best reflect the state of your health TODAY. (Russia and Belarus only)

Display This Question:

If Q2 = 143

Or Q2 = 16

Q142 What would you say is your mobility and ability to move around TODAY? (Russian and Belarus only)

- ◯ I experience no difficulties in mobility (4)
- ◯ I experience some difficulties in mobility (7)
- ◯ I am bedridden (8)

Display This Question:

If Q2 = 143

Or Q2 = 16

Q143 What would you say is your ability to take care of yourself TODAY: (Russia and Belarus only)

- ◯ I experience no difficulties in caring for myself (4)
- ◯ I experience some difficulties in washing or dressing myself (9)
- ◯ I am not able to wash or dress myself (10)

Display This Question:

If Q2 = 143

Or Q2 = 16

Q144 What would you say about your daily activities (e.g. work, study, housework, family business, entertainment) TODAY: (Russia and Belarus only)

- ◯ I do not experience any difficulties in my normal daily activities (4)
- ◯ I experience some difficulties in my normal daily activities (11)
- ◯ I am not able to engage in my normal daily activities (12)

Display This Question:

If Q2 = 143

Or Q2 = 16

Q145 What would you say about your feeling of pain or discomfort TODAY: (Russia and Belarus only)

- ◯ I am not experiencing any pain or discomfort (16)
- ◯ I am experiencing manageable pain or discomfort (19)
- ◯ I am experiencing severe pain or discomfort (20)

Display This Question:

If Q2 = 143

Or Q2 = 16

Q146 What would you say about your feeling of anxiety or depression TODAY: (Russia and Belarus only)

- ◯ I am not experiencing any anxiety or depression (16)
- ◯ I am experiencing moderate anxiety or depression (22)
- ◯ I am experiencing severe anxiety or depression (23)

Display This Question:

If Q2 = 16

Or Q2 = 143

Or Q2 = 183

Q140

Before you is a scale from 0 to 100. 100 (right on the scale) represents the very **best** state of health which you can imagine, 0 (left on the scale) is the absolute **worst** state of health that you can imagine.

0 5 101520253035404550556065707580859095100

Choose the point that reflects your health today. ()	

End of Block: Russian version EQ5D

Start of Block: Coronavirus - self infected

Q44 Have you ever had, or suspected you might have, the Coronavirus (COVID-19)?

◯ No (2)

◯ Yes (1)

◯ Unsure (3)

◯ Prefer not to Answer (4)

Display This Question:

If Q44 = 1

Q45 Which of the following best describes your situation?

▼ I have not been tested (1) ... Other (specify) (6)

Q46 If I wanted to get a **COVID-19 test**, I could do this at (select all that apply)

	With COVID-19 symptoms (1)	Without COVID-19 symptoms (2)
Public clinic or hospital (1)	▢	▢
Private clinic or hospital (4)	▢	▢
Pharmacy (5)	▢	▢
At my workplace (12)	▢	▢
Self-test (6)	▢	▢
Only if life-threatening symptoms (9)	▢	▢
If getting treatment for other conditions (10)	▢	▢
Would not be able to be tested (8)	▢	▢
Don't know (7)	▢	▢

Q47 Do you regularly update your vaccinations for diseases present in your area?

○ No (3)

○ Some of them (2)

○ Yes (1)

Display This Question:

If Q11 = 1 [1]

Or Q11 = 1 [2]

Q48 Do you follow the recommended schedule of vaccines for your child(ren)?

- No (3)
- Some of them (2)
- Yes (1)

Q49 If a vaccine for COVID-19 was made available to you at no cost, would you get it?

- No (3)
- Maybe (2)
- Yes (1)

End of Block: Coronavirus - self infected

Start of Block: Coronavirus - other infected

Q50 Has anyone you know ever been suspected or confirmed to have Coronavirus (COVID-19)?

- No (2)
- Yes (1)
- Unsure (3)
- Prefer not to Answer (4)

End of Block: Coronavirus - other infected

Start of Block: Coronavirus - Risk and Control/God scale

Page Break

Q51 The following questions include beliefs about **how much control** we have over our likelihood of becoming ill with COVID-19. Please state how much you agree with each statement.

	Strongly disagree (8)	Somewhat disagree (9)	Somewhat agree (11)	Strongly agree (12)
I have very little control over whether or not I become ill with COVID-19. (5)	○	○	○	○
Whether or not I become seriously ill is up to fate. (1)	○	○	○	○
Whatever happens is God's will. (4)	○	○	○	○

Q52 To what extent are you concerned about **your personal risk** of being infected with COVID-19?

▼ Not at all (1) ... Extremely (4)

Q53 To what extent are you concerned about **your loved ones** being infected with COVID-19?

▼ Not at all (1) ... Extremely (4)

Page Break

Q54 I am concerned that if I were infected with COVID-19, it may create **a great burden** for my family.

▼ Not at all (1) ... Extremely (5)

Q55 I am concerned that if I were infected with COVID-19, it may cause others to devalue or **discriminate against me**.

▼ Not at all (1) ... Extremely (5)

End of Block: Coronavirus - Risk and Control/God scale

Start of Block: Trust - Generalized (used in US Generalized Social Survey, 1972-2018)

Q56 The next set of questions ask about how much trust and confidence you have in people and in various organizations.

Page Break

Q57 Generally speaking, would you say that most people can be trusted, or that you can’t be too careful in dealing with people?

○ most people can be trusted (1)

○ you can't be too careful in dealing with people (2)

Q58 Do you think that most people would try to take advantage of you if they got the chance, or would they try to be fair?

○ try to take advantage (1)

○ try to be fair (4)

Q59 Would you say that most of the time people try to be helpful or that they are mostly looking out for themselves?

○ try to be helpful (1)

○ mostly looking out for themselves (2)

End of Block: Trust - Generalized (used in US Generalized Social Survey, 1972-2018)

Start of Block: Trust - Types of People

Q60 In general, **how much do you trust** each of the following groups of people?

	Do not trust at all (1)	Do not trust very much (2)	Trust somewhat (3)	Trust completely (4)
People in your family? (1)	○	○	○	○
People in your community? (13)	○	○	○	○
People you work or go to school with? (14)	○	○	○	○
People who speak a different language? (15)	○	○	○	○
People of another nationality? (16)	○	○	○	○
People of another religion? (17)	○	○	○	○
Doctors, nurses and other medical practitioners? (19)	○	○	○	○
Religious leaders? (12)	○	○	○	○
Scientists? (21)	○	○	○	○
Politicians? (22)	○	○	○	○
Journalists? (23)	○	○	○	○

Page Break

Q61 In general, **how much confidence** do you have in the following organizations or individuals information about and handling of the Coronavirus?

	None at all (1)	Not very much (2)	Some confidence (3)	A lot of confidence (5)
World Health Organization (WHO) (1)	○	○	○	○
My country's national health organization or Ministry of Health (e.g., CDC, PHAC) (2)	○	○	○	○
Non-governmental aid organizations (NGOs) (17)	○	○	○	○
Religious organizations (12)	○	○	○	○
Religious leaders (13)	○	○	○	○
Q2 != 1 And Q2 != 36 Top leader of my country's government (18)	○	○	○	○
US President (4)	○	○	○	○
Q2 != 36 China's President (5)	○	○	○	○
Q2 != 143 And Q2 != 16 My national government (16)	○	○	○	○
Q2 = 1 My governor (19)	○	○	○	○
Q2 = 31 Or Q2 = 36 Or Q2 = 79 My provincial leader (20)	○	○	○	○

Q2 != 1<br>*And Q2 != 31*<br>*And Q2 != 36*<br>*And Q2 != 79*<br>*And Q2 != 179*<br>*And Q2 != 143*<br>*And Q2 != 16*<br>My district or regional government (21)	○	○	○	○
My local government (7)	○	○	○	○
My local health department (10)	○	○	○	○
My employer (11)	○	○	○	○

Page Break

End of Block: Trust - Types of People

Start of Block: Attribution of China and US

Q62 If you believe that "China" should be blamed for the COVID-19 pandemic, who specifically do you blame? (Check all that apply)

- ☐ The Chinese government (1)
- ☐ The Chinese Communist Party (3)
- ☐ President Xi Jinping (5)
- ☐ The Wuhan municipal goverment (4)
- ☐ Chinese society and people (6)
- ☐ ⊗I don't blame China. (7)

Page Break

Q63 If you believe that **"the U.S."** should be blamed for the COVID-19 pandemic, who specifically do you blame? (Check all that apply)

- ▢ The U.S. government (1)
- ▢ The U.S. Center for Disease Control (CDC) (2)
- ▢ President Trump (3)
- ▢ State and local governments (5)
- ▢ American society and people (4)
- ▢ I don't blame the U.S. (6)

End of Block: Attribution of China and US

Start of Block: Responsibility with Prompts

Q64 **The following section includes information that has been published in the popular press. Please read the following paragraph and respond to how much you agree with the statements listed below.**

Q65 COVID-19, the Coronavirus disease we’re asking about in this survey, is a new disease that has not been previously identified in humans. It was discovered in late 2019 and is currently spreading throughout the world. Symptoms of the Coronavirus (COVID-19) can range from mild to severe, including fever, cough, difficulty breathing, pneumonia, and, in severe cases, death.

Q66 China has scored a great victory in combatting COVID-19. The lockdown of the city of Wuhan and Hubei province on January 23 turned out to be a forward-thinking and decisive move in helping curb the spread of COVID-19. The WHO has spoken highly of China's contribution to disease prevention and control. In contrast, the vast majority of countries failed to mobilize their people fast enough to contain the virus.

Q67 China intentionally lied to the world about the danger of COVID-19. It prevented a global response that might have stopped it from becoming a pandemic. It would have been much better if the world had known about the coronavirus outbreak much earlier. Instead, China's cover-up hampered other countries' efforts to prepare for the arrival of the virus. The world is paying a very big price for what China did.

Page Break

Q68

How much do you agree with the following statements about who is responsible for the COVID-19 pandemic.

	Disagree (1)	Somewhat disagree (2)	Somewhat Agree (3)	Agree (4)
China is responsible (1)	o	o	o	o
The U.S. is responsible (5)	o	o	o	o
Italy is responsible (6)	o	o	o	o
The WHO (World Health Organization) is responsible (7)	o	o	o	o
Countries that failed to prepare for an outbreak are responsible (8)	o	o	o	o
People who do not follow social distancing guidelines are responsible (13)	o	o	o	o
The U.S. military planted the COVID-19 virus in China. (9)	o	o	o	o
The COVID-19 virus originated in a Chinese lab. (10)	o	o	o	o
China should compensate other countries for damages caused by the pandemic. (11)	o	o	o	o

End of Block: Responsibility with Prompts

Start of Block: Big 5 Personality

Q69 The next few questions ask about **how you see yourself** and what you value in yourself and other people.

Q70 I see myself as...

	Disagree strongly (1)	Disagree a little (3)	Agree a little (5)	Agree strongly (7)
Extroverted, enthusiastic (1)	○	○	○	○
Critical, quarrelsome (2)	○	○	○	○
Dependable, self-disciplined (3)	○	○	○	○
Anxious, easily upset (4)	○	○	○	○
Open to new experiences, creative (5)	○	○	○	○

End of Block: Big 5 Personality

Start of Block: Discrimination & Race/Ethn (survey experiment - randomize order of these two Qs?

Q71 Compared to **before** the Coronavirus pandemic, how often do these things happen to you?

	Less (1)	Same (2)	More (3)
Treated with courtesy or respect (1)	○	○	○
Received poorer service than others (2)	○	○	○
Threatened or harassed (4)	○	○	○
Strangers displayed negative reactions to you (5)	○	○	○

Page Break

Display This Question:

If Q2 = 1

Q72 Indicate your race/ethnic identity (check all that apply)

- ▢ Arabic/Middle Eastern (3)
- ▢ Black/African (2)
- ▢ Caribbean (4)
- ▢ Caucasian/White European (1)
- ▢ Central Asian (13)
- ▢ East Asian (Chinese, Japanese, Korean) (5)
- ▢ Latino/Mexican/Central/South American (9)
- ▢ Native American (14)
- ▢ South Asian (India, Sri Lanka, Pakistan) (7)
- ▢ South East Asian (Vietnamese, Malaysian, etc.) (6)
- ▢ Pacific Islander (8)
- ▢ Multi-racial/multicultural (10)
- ▢ Other (11) ____

Display This Question:

If Q2 = 36

Q73 Indicate your race/ethnic identity (check all that apply)

- ▢ Han (3)
- ▢ Zhuang (2)
- ▢ Hui (15)
- ▢ Manchu (16)
- ▢ Uyghur (17)
- ▢ Other (11) ____

Page Break

Display This Question:

If Q2 = 143

Or Q2 = 16

Or Q2 = 183

Q138 What is your ethnicity? (Russia, Ukraine, Belarus)

____

Q74 What is your current religion, if any? (Select all that apply)

- [ ] Atheist or agnostic (11)
- [ ] Buddhist (9)
- [ ] Catholic (4)
- [ ] Christian/Protestant/Methodist/Lutheran/Baptist (1)
- [ ] Greek or Russian Orthodox (6)
- [ ] Jewish (7)

Q2 != 143

And Q2 != 16

And Q2 != 183

- [ ] Hindu (10)
- [ ] Mormon (5)
- [ ] Muslim (8)
- [ ] Nothing in particular (12)
- [ ] Other (13) ____

Q75 How **important** is religion in your life?

▼ Not at all (1) ... Extremely important (4)

Page Break

Q76 Thinking of **religion**, how serious is the conflict between people who are very religious and people who are **not** very religious in your country?

▼ Very serious (1) ... Do not wish to answer (8)

Q77 Do you think this conflict has changed since the COVID-19 outbreak?

▼ Yes, it has gotten much worse (1) ... Yes, it has gotten much better (7)

End of Block: Discrimination & Race/Ethn (survey experiment - randomize order of these two Qs?

Start of Block: Demographics - personal

Q78 The following questions are basic demographic questions to help us understand how people around the world who share your characteristics think similarly or differently from you. You are not required to answer these questions, but doing so helps us understand whether there are similarities in how people respond based on these characteristics, or whether the current situation of the pandemic is a better explanation for how people make decisions about their own behavior in response to the pandemic.

Q80 What is the highest level of education you have completed?

▼ Less than high school (1) ... Currently studying (Russia/Belarus/Ukrainian) (7)

Display This Question:

If If What is your year of birth? Text Response Is Greater Than or Equal to 1996

Q81 Father's educational level

▼ Less than high school (1) ... Doctoral degree (Ph.D.) (6)

Display This Question:

If If What is your year of birth? Text Response Is Greater Than or Equal to 1996

Q82 Mother's educational level

▼ Less than high school (1) ... Doctoral degree (Ph.D.) (6)

End of Block: Demographics - personal

Start of Block: Demographics - political

Display This Question:

If Q2 = 172

Or Q9 = 172

Q83 Which of these parties did you support in the last election? (Thailand)

▢ Palang Pracharat Party (1)

▢ Pheu Thai Party (9)

▢ Future Forward Party (10)

▢ Did not vote (8)

▢ Other (7) ____

Display This Question:

If Q2 = 136

Or Q9 = 136

Q84 Which presidential candidate did you support in the 2016 election? (the Philippines)

- ◯ Rodrigo Duterte (1)
- ◯ Mar Roxas (4)
- ◯ Grace Poe (5)
- ◯ Did not vote (9)
- ◯ Other (8) ____

Display This Question:

If Q2 = 79

Or Q9 = 79

Q85 Which presidential candidate did you support in the last election? (Indonesia)

- ◯ Joko Widodo (1)
- ◯ Prabowo Subianto (4)
- ◯ Did not vote (5)

Display This Question:

If Q2 = 104

Or Q9 = 104

Q86 Which party do you most closely identify with? (Malaysia)

- ◯ Bersatu (1)
- ◯ Parti Keadilan Rakyat (4)
- ◯ UMNO (5)
- ◯ PAS (6)
- ◯ Other (7) ____

Display This Question:

If Q2 = 118

Or Q9 = 118

Q87 Which of these parties did you support in the last election? (Myanmar)

- ▢ NLD (1)
- ▢ USDP (4)
- ▢ Arakan National Party (5)
- ▢ Did not vote (7)
- ▢ Other (6) ____

Display This Question:

If Q2 = 1

Or Q9 = 1

Q88 Who do you expect to vote for in the 2020 presidential election? (U.S.)

- ◯ Donald Trump (1)
- ◯ Joe Biden (4)
- ◯ Unsure (6)
- ◯ Will not vote (7)
- ◯ Other (5) ____

Display This Question:

If Q2 = 31

Or Q9 = 31

Q89 Which of these parties did you support in the last election? (Canada)

- ◯ Liberal Party (1)
- ◯ Conservative Party (4)
- ◯ New Democratic Party (5)
- ◯ Did not vote (7)
- ◯ Other (6) ____

Display This Question:

If Q2 = 173

Or Q9 = 173

Q90 Which presidential candidate did you support in the last election? (Taiwan)

- ◯ Tsai Ing-wen (1)
- ◯ Han Kuo-yu (4)
- ◯ Did not vote (5)
- ◯ Other (6) ____

Display This Question:

If Q2 = 156

Or Q9 = 156

Q91 Which of these parties did you support in the last election? (Singapore)

- ◯ People's Action Party (1)
- ◯ Workers' Party (4)
- ◯ Did not vote (6)
- ◯ Other (5) ____

Display This Question:

If Q2 = 168

Or Q9 = 168

Q92 Which of these parties did you support in the last election? (Sweden)

◯ Social Democratic Party (1)

◯ Moderate Party (4)

◯ Sweden Democrats (5)

◯ Did not vote (7)

◯ Other (6) ____

Display This Question:

If Q2 = 137

Or Q9 = 137

Q93 Which of these parties did you support in the last parliamentary election? (Poland)

◯ Law and Justice Party (1)

◯ Civic Coalition (4)

◯ The Left (5)

◯ Did not vote (7)

◯ Other (6) ____

End of Block: Demographics - political

Start of Block: Demographics - Income and Employment

Q94 Which statement best describes your current employment status? (check all that apply)

- ☐ Working full-time in job I held prior to COVID-19 crisis (9)
- ☐ Working part-time in job I held prior to COVID-19 crisis (2)
- ☐ Lost a job or hours because my employer shut down/downsized due to COVID-19 (3)

Q2 = 16
Or Q2 = 143
Or Q2 = 183

- ☐ My hours were cut (18)

Q2 = 16
Or Q2 = 143
Or Q2 = 183

- ☐ My pay was cut (19)
- ☐ Left a job because I did not think it safe to work during the COVID-19 crisis (4)
- ☐ Working full-time in a job I started during COVID-19 (7)
- ☐ Working part-time in a job I started during COVID-19 (8)
- ☐ Working in 'gig' economy (e.g., ride share, food delivery, etc.) (5)
- ☐ Currently unemployed and looking for work (12)
- ☐ Currently unemployed and not looking for work (14)
- ☐ Housekeeping/caring for my family as my main job (15)
- ☐ Retired (1)
- ☐ Student (6)
- ☐ Self-employed (16)

- [ ] Small business owner (17)

Q95 What measures, if any, has your government enacted to financially support individuals or businesses during the COVID-19 pandemic? (Select all that apply)

- [ ] Large companies compensated for losses (1)
- [ ] Small companies compensated for losses (2)
- [ ] Companies offered loans (3)
- [ ] Increased unemployment insurance (4)
- [ ] Government subsidies for individuals (5)
- [ ] One time stimulus checks (6)
- [ ] Free testing (7)
- [ ] None (9)
- [ ] Don't Know (10)
- [ ] Other (8) ____

Q96 Which of the following statements most accurately reflects the financial situation of your family **before** COVID-related policies took effect?

- ◯ We do not have enough money for food. (1)
- ◯ We have enough money for food, but not enough money for clothes. (4)
- ◯ We have enough money to buy food and clothes, but not enough to buy expensive items, such as a TV or refrigerator. (5)
- ◯ We have expensive items, such as a new TV or refrigerator, but no car. (6)
- ◯ We can buy almost anything we want. (7)

Display This Question:

If Q2 = 1

Q97 Annual family income (U.S.)

- Under $15,000 (1)
- $15,000-$19,999 (4)
- $20,000-$24,999 (5)
- $25,000-$29,999 (6)
- $30,000-$34,999 (7)
- $35,000-$39,999 (8)
- $40,000-$44,999 (9)
- $45,000-$49,999 (10)
- $50,000-$54,999 (11)
- $55,000-$59,999 (12)
- $60,000-$64,999 (13)
- $65,000-$69,999 (14)
- $70,000-$74,999 (15)
- $75,000-$79,999 (16)
- $80,000-$84,999 (17)
- $85,000-$89,999 (18)
- $90,000-$94,999 (19)
- $95,000-$99,999 (20)
- $100,000-$124,999 (21)
- $125,000-$149,999 (22)
- $150,000-$199,999 (23)
- $200,000+ (24)

Display This Question:

If Q2 = 156

Q98 Annual family income (Singapore)

- Less than 30,000 SGD (1)
- 30,000 to 39,999 SGD (4)
- 40,000 to 49,999 SGD (5)
- 50,000 to 59,999 SGD (6)
- 60,000 to 69,999 SGD (7)
- 70,000 to 79,999 SGD (8)
- 80,000 to 89,999 SGD (9)
- 90,000 to 99,999 SGD (10)
- 100,000 to 129,999 SGD (11)
- 130,000 to 159,999 SGD (12)
- 160,000 or more (13)

Display This Question:

If Q2 = 75

Q99 Annual family income (Hong Kong)

- < 125 000 HKD (1)
- 125 000 - 159 999 HKD (4)
- 160 000 - 189 999 HKD (5)
- 190 000 - 254 999 HKD (6)
- 255 000 - 319 999 HKD (7)
- 320 000 - 379 999 HKD (8)
- 380 000 - 444 999 HKD (9)
- 445 000 - 508 499 HKD (10)
- 508 500 - 569 999 HKD (11)
- 570 000 - 634 999 HKD (12)
- 635 000 or more (13)

Display This Question:

If Q2 = 173

Q100 Annual family income (Taiwan)

◯ (1)

◯ 500,000 - 650,000 New Taiwan Dollars (4)

◯ 650,000 - 775,000 New Taiwan Dollars (5)

◯ 775,000 - 1,000,000 New Taiwan Dollars (6)

◯ 1,000,000 - 1,300,000 New Taiwan Dollars (7)

◯ 1,300,000 - 1,550,000 New Taiwan Dollars (8)

◯ 1,550,000 - 1,800,000 New Taiwan Dollars (9)

◯ 1,800,000 - 2,050,000 New Taiwan Dollars (10)

◯ 2,050,000 - 2,300,000 New Taiwan Dollars (11)

◯ 2,300,000 - 2,600,000 New Taiwan Dollars (12)

◯ >2,600,000 New Taiwan Dollars (13)

Display This Question:

If Q2 = 192

Q101 Monthly family income (Viet Nam)

- 0 - 1 499 999 VND (monthly) (1)
- 1 500 000 VND - 2 999 999 VND (monthly) (4)
- 3 000 000 VND - 4 499 999 VND (monthly) (5)
- 4 500 000 VND - 5 499 999 VND (monthly) (6)
- 5 500 000 VND - 6 499 999 VND (monthly) (7)
- 6 500 000 VND - 7 499 999 VND (monthly) (8)
- 7 500 000 VND - 8 499 999 VND (monthly) (9)
- 8 500 000 VND - 9 499 999 VND (monthly) (10)
- 9 500 000 VND - 10 499 999 VND (monthly) (11)
- 10 500 000 VND - 11 499 999 VND (monthly) (12)
- 11 500 000 VND - 12 499 999 VND (monthly) (13)
- 12 500 000 VND - 13 499 999 VND (monthly) (14)
- 13 500 000 VND - 14 999 999 VND (monthly) (15)
- 15 000 000 VND - 29 999 999 VND (monthly) (16)
- 30 000 000 VND - 44 999 999 VND (monthly) (17)
- 45 000 000 VND - 74 999 999 VND (monthly) (18)
- 75 000 000 VND - 149 999 999 VND (monthly) (19)
- 150 000 000 VND or higher (monthly) (20)

Display This Question:

If Q2 = 179

Q102 Monthly family income (Turkey)

- 0-1000 TL (1)
- 1000-1500 TL (10)
- 1500-2000 TL (11)
- 2000-2500 TL (12)
- 2500-3000 TL (13)
- 3000-5000 TL (14)
- 5000-7000 TL (15)
- 7000-10000 TL (16)
- 10000-15000 TL (17)
- Above 15000 TL (18)

Display This Question:

If Q2 = 136

Q103 Annual family income (the Philippines)

- Below PHP 30,000 (1)
- PHP 30,000 to 60,000 (4)
- PHP 60,001 to 90,000 (5)
- PHP 90,001 to 120,000 (6)
- PHP 120,001 to 150,000 (7)
- PHP 150,001 to 180,000 (8)
- PHP 180,001 to 210,000 (9)
- PHP 210,001 to 240,000 (10)
- PHP 240,001 to 270,000 (11)
- PHP 270,001 to 300,000 (12)
- PHP 300,001 to 360,000 (13)
- PHP 360,001 to 420,000 (14)
- PHP 420,001 to 480,000 (15)
- PHP 480,001 and above (16)

Display This Question:

If Q2 = 168

Q104 Annual family income (Sweden)

- Less than 65 000 SEK (1)
- 65 000 - 129 999 SEK (4)
- 130 000 - 194 999 SEK (5)
- 195 000 - 259 999 SEK (6)
- 260 000 - 324 999 SEK (7)
- 325 000 - 389 999 SEK (8)
- 390 000 - 519 999 SEK (9)
- 520 000 - 649 999 SEK (10)
- 650 000 - 779 999 SEK (11)
- 780 000 - 1 039 999 SEK (12)
- 1 040 000 - 1 299 999 SEK (13)
- 1 300 000 and more (14)

Display This Question:

If Q2 = 172

Q105 Annual family income (Thailand)

- Less than 70,000 Baht (1)
- 70,001 - 135,000 Baht (4)
- 135,001 - 200,000 Baht (5)
- 200,001 - 265,000 Baht (6)
- 265,001 - 330,000 Baht (7)
- 330,001 - 395,000 Baht (8)
- 395,001 - 460,000 Baht (9)
- 460,001 - 525,000 Baht (10)
- 525,001 - 590,000 Baht (11)
- 590,001 - 655,000 Baht (12)
- 655,001 - 900,000 Baht (13)
- 900,001 - 1,200,000 Baht (14)
- 1,200,001 - 1,500,000 Baht (15)
- 1,500,001 - 1,800,000 Baht (16)
- 1,800,001 - 2,100,000 Baht (17)
- 2,100,001 - 2,400,000 Baht (18)
- 2,400,001 - 2,700,000 Baht (19)
- 2,700,001 - 3,000,000 Baht (20)
- 3,000,000 Baht or more (21)

Display This Question:

If Q2 = 79

Q106 Annual family income (Indonesia)

- Below Rp. 5 million (1)
- From Rp. 5 - 10 million (4)
- From Rp. 10 - 15 million (5)
- From Rp. 15 - 20 million (6)
- From Rp. 20 - 25 million (7)
- From Rp. 25 - 30 million (8)
- From Rp. 30 - 35 million (9)
- From Rp. 35 - 40 million (10)
- From Rp. 40 - 45 million (11)
- From Rp. 45 - 50 million (12)
- From Rp. 50 - 55million (13)
- From Rp. 55 - 60 million (14)
- From Rp. 60 - 65 million (15)
- From Rp. 65 - 70 million (16)
- From Rp. 70 - 75 million (17)
- From Rp. 75 - 80 million (18)
- From Rp. 80 - 85 million (19)
- From Rp. 85 - 90 million (20)
- From Rp. 90 - 95 million (21)
- From Rp. 95 - 100 million (22)
- More than Rp. 100 million (23)
- More than Rp. 500 million (24)

Display This Question:

If Q2 = 104

Q107 Annual family income (Malaysia)

- Less than 20,000 Ringgit (1)
- 20,000 - 34,999 Ringgit (4)
- 35,000 - 49,999 Ringgit (5)
- 50,000 - 99,999 Ringgit (6)
- 100,000 Ringgit or more (7)

Display This Question:

If Q2 = 36

Q108 Annual family income (mainland China)

- < 20'000 CNY (1)
- 20'000-29'999 CNY (4)
- 30'000-49'999 CNY (5)
- 50'000-69'999 CNY (6)
- 70'000-99'999 CNY (7)
- 100'000-119'999 CNY (8)
- 120'000-129'999 CNY (9)
- 130'000-149'999 CNY (10)
- 150'000-179'999 CNY (11)
- 180'000-199'999 CNY (12)
- 200'000-219'000 CNY (13)
- 220'000-239'999 CNY (14)
- 240'000- 269'999CNY (15)
- 270'000-299'999CNY (16)
- > 300'000 CNY (17)

Display This Question:

If Q2 = 31

Q109 Annual family income (Canada)

- Under $15,000 (1)
- $15,000-$19,999 (4)
- $20,000-$24,999 (5)
- $25,000-$29,999 (6)
- $30,000-$34,999 (7)
- $35,000-$39,999 (8)
- $40,000-$44,999 (9)
- $45,000-$49,999 (10)
- $50,000-$54,999 (11)
- $55,000-$59,999 (12)
- $60,000-$64,999 (13)
- $65,000-$69,999 (14)
- $70,000-$74,999 (15)
- $75,000-$79,999 (16)
- $80,000-$84,999 (17)
- $85,000-$89,999 (18)
- $90,000-$94,999 (19)
- $95,000-$99,999 (20)
- $100,000-$124,999 (21)
- $125,000-$149,999 (22)
- $150,000-$199,999 (23)
- $200,000+ (24)

Display This Question:

If Q2 = 16

Or Q2 = 143

Or Q2 = 183

Q139 What is your monthly income in your national currency)? (Belarus, Russia, Ukraine)

____

End of Block: Demographics - Income and Employment

Start of Block: Additional concerns then link to draw with email entered in separate survey

Page Break

Q110 Please let us know if there are any additional concerns or thoughts you might have regarding the current Coronavirus crisis and how people make decisions about their own and others' safety.

____

____

____

____

____

End of Block: Additional concerns then link to draw with email entered in separate survey

---

Start of Block: Thank you for participating in our study!

Q112 **Thank you for participating in our study!**
We appreciate your participation in helping us understand how COVID-19 is affecting you and your family.

According to current health information, COVID-19 is a highly contagious virus that affects different people in different ways. Most infected people will develop mild to moderate symptoms. Common symptoms include: fever, tiredness, and dry cough. In addition, some people may experience: aches and pains, nasal congestion, runny nose, sore throat, and diarrhoea. If you would like more information about COVID-19, including how to prevent and treat infections, please contact your local health agency, the U.S. Centers for Disease Control, the Public Health Agency of Canada, or the WHO website.
If you would like to see factors that can affect the spread of diseases like COVID-19, please click here.

If you have questions about your rights as a research participant, or wish to obtain information, ask questions or discuss any concerns about this study with someone other than the researcher(s), please contact the University of Michigan Health Sciences and Behavioral Sciences Institutional Review Board (IRB-HSBS) , 2800 Plymouth Rd., Building 520, Room 1169 , Ann Arbor, MI 48109-2800 , Phone: 734-936-0933 , Fax: 734-936-1852 , Email: irbhsbs@umich.edu. eResearch ID: HUM00168677

Please contact us if you would be willing to participate in a follow-up questionnaire or interview: covid.decisions@umich.edu

Appendix A Rescoring Table

Original Question(s)	New Variable	Calculation	# of Items	Min - Max
Q14 concerns about children	concerns about children	1 point/item;<br>0 for "none"	8	0 - 7
Q16 Gov_restriction	COVID Prevention government actions - current	1 point/item	22	0-22
	COVID Prevention government actions - past	1 point/item	22	0-22
Q17 Self actions	COVID Prevention self-action	1 point/item;<br>0 for no action	16	0-15
Q26 COVID_difficulties	COVID difficulties	1 point/item;<br>0 for no difficult	15	0-14
Q27 COVID_impact	COVID impact	-2, -1, 1, 2	8	Min = -16<br>Max = 16
Q34 Statements about COVID-19	COVID fact	For item (1), (15), (14)<br>Disagree = 1, agree = -1<br>For item (2), (8), (10), (13), (14), (16)<br>Disagree = -1, agree = 1	9 = (1) + (2) +(15) + (4) +(8)+ (10) + (13) + (14) + (16)	Min = -9 (knowing<br>Max = 9 (knowing
	COVID conspiracy	Disagree = -1<br>Agree = 1	4 = (19) + (18) +(5) +(11)	Min = -4 (Don't be<br>all)<br>Max = 4 (believe<br>much)
	COVID suspicion	For item (6), (17)<br>Disagree = -1, agree = 1<br>For item (9)<br>disagree = 1, agree = -1	3 = (6) + (9) + (17)	Min = -3 (not susp<br>Max = 3 (suspicio
Q36 Emotions	Past month – positive emotion	Less = -1<br>Same = 0<br>More = 1	3 = (19) + (20) + (21)	Min = -3 (positive e<br>Max = 3 (positive e
	Past month – positive emotion	Less = -1<br>Same = 0<br>More = 1	4	Min = -4 (negative<br>Max = 4 (negative e
Q37 Ways of coping	Past month – ways to cope	Less = -1<br>Same = 0<br>More = 1	18	Min = -18<br>Max = 18
Q40 Health problems	Health Problems	1 point/item except for "other" and "prefer not to answer"<br>0 points for "none"	11	Min = 0<br>Max = 11

Q42 Physical symptoms	Past month—physical symptoms	No = 0<br>Somewhat = 1<br>Often = 2	7	Min = 0<br>Max = 14
Q43 COVID symptoms	Health-symptom	1 piont/item<br>No = 0	9	Min = 0<br>Max = 8
Q 51 how much control you have	Risk and control	Strongly disagree = 2<br>Somewhat disagree = 1<br>Somewhat agree = -1<br>Strongly agree = -2	3	Min = -6 (have co[illegible]<br>Max = 6 (no contr[illegible]
Q57, 58, 59 Trust-general	Trust-generalized	Q57-(1) = 1<br>Q57-(2) = -1<br>Q58-(1) = -1<br>Q58-(4) = 1<br>Q59-(1) = 1<br>Q59-(2) = -1	3	Min = -3 (Don't tru[illegible]<br>Max = 3 (trust in [illegible]
Q60 Trust people	Trust-people	Do not trust at all = -2<br>Do not trust very much = -1<br>Trust somewhat = 1<br>Trust completely = 2	11	Min = -22 (don't tr[illegible]<br>Max = 22 (trust co[illegible]
Q61 Trust-organization	Trust-organization	None at all = -2<br>Not very much = -1<br>Some confidence = 1<br>A lot of confidence = 2	15	Min = -30 (no con[illegible]<br>Max = 30 (lots of [illegible]
Q62 China blame	China blame	1 point/item	5	Min = 0 (don't bla[illegible]<br>Max = 5 (blame C[illegible]
Q63 US blame	US_blame	1 point/item	5	Min = 0 (don't bla[illegible]<br>Max = 5 (blame th[illegible]
Q68 who is responsible	China_responsible	Disagree = -2<br>Somewhat disagree = -1<br>Somewhat agree = 1<br>Agree = 2	3 = (1) + (10) + (11)	Min = -6 (China is n[illegible]<br>Max = 6 (China is re[illegible]
	US_responsible	Disagree = -2<br>Somewhat disagree = -1<br>Somewhat agree = 1<br>Agree = 2	2 = (5) + (9)	Min = -4 (US is not [illegible]<br>Max = 4 (US is resp[illegible]
	Other_responsible	Disagree = -2<br>Somewhat disagree = -1<br>Somewhat agree = 1<br>Agree = 2	4 = (6) + (7) + (8) + (13)	Min = -8 (Other is n[illegible]<br>Max = 8 (Other is re[illegible]
Q70 Big 5 personality	Big5_openess	Disagree strongly = -2<br>Disagree a little = -1<br>Agree a little = 1<br>Agree strongly = 2	1= (5)	Min = -2 (low end[illegible]<br>Max = 2 (high end[illegible]
	Big5_conscientiousness	Disagree strongly = -2<br>Disagree a little = -1<br>Agree a little = 1<br>Agree strongly = 2	1 = (3)	Min = -2 (low end[illegible]<br>Max = 2 (high end[illegible]

	Big5_extroversion	Disagree strongly = -2<br>Disagree a little = -1<br>Agree a little = 1<br>Agree strongly = 2	1 = (1)	Min = -2 (low end [illegible]<br>Max = 2 (high en[illegible]
	Big5_agreeableness	Disagree strongly = 2<br>Disagree a little = 1<br>Agree a little = -1<br>Agree strongly = -2	1 = (2)	Min = -2 (low end [illegible]<br>Max = 2 (high en[illegible]
	Big5_neuroticism	Disagree strongly = -2<br>Disagree a little = -1<br>Agree a little = 1<br>Agree strongly = 2	1 = (4)	Min = -2 (low end [illegible]<br>Max = 2 (high en[illegible]
